# Cytotoxic phenanthroline derivatives alter metallostasis and redox homeostasis in neuroblastoma cells

**DOI:** 10.18632/oncotarget.26346

**Published:** 2018-11-20

**Authors:** Irina Naletova, Cristina Satriano, Alessandra Curci, Nicola Margiotta, Giovanni Natile, Giuseppe Arena, Diego La Mendola, Vincenzo Giuseppe Nicoletti, Enrico Rizzarelli

**Affiliations:** ^1^ Department of Chemical Sciences, University of Catania, Catania, Italy; ^2^ Consorzio Interuniversitario di Ricerca in Chimica dei Metalli nei Sistemi Biologici (CIRCMSB), Bari, Italy; ^3^ Department of Chemistry, University of Bari ‘Aldo Moro’, Bari, Italy; ^4^ Department of Pharmacy, University of Pisa, Pisa, Italy; ^5^ Section of Medical Biochemistry, Department of Biomedical and Biotechnological Sciences (BIOMETEC), University of Catania, Catania, Italy

**Keywords:** metal homeostasis, anticancer drug, ionophores, SH-SY5Y cell line, oxidative stress

## Abstract

Copper homeostasis is generally investigated focusing on a single component of the metallostasis network. Here we address several of the factors controlling the metallostasis for neuroblastoma cells (SH-SY5Y) upon treatment with 2,9-dimethyl-1,10-phenanthroline-5,6-dione (phendione) and 2,9-dimethyl-1,10-phenanthroline (cuproindione). These compounds bind and transport copper inside cells, exert their cytotoxic activity through the induction of oxidative stress, causing apoptosis and alteration of the cellular redox and copper homeostasis network. The intracellular pathway ensured by copper transporters (Ctr1, ATP7A), chaperones (CCS, ATOX, COX 17, Sco1, Sco2), small molecules (GSH) and transcription factors (p53) is scrutinised.

## INTRODUCTION

The widespread success of cisplatin (cis-diamino-dichloroplatinum(II)) [1] (cisplatin) for the clinical treatment of various types of cancers in the last 40 years has placed the coordination chemistry of metal-based drugs at the front line in the battle against cancer. The toxic side effects, the low bioavailability and the innate or developed drug resistance of the first FDA-approved platinum drug have only partially been overcome by second and third generation platinum compounds [2]. These issues have stimulated the development of alternative anticancer drugs based on other noble metals [3–6]. Among the first row transition metal complexes, copper coordination compounds are the most promising metal-based anticancer/cytotoxic agents [7]. Copper alteration in cancer has been studied for several decades and aberrant levels of this metal have been found in malignant tissues of both tumour bearing mice and patients suffering from cancer [8–11]. Additionally, high serum concentrations of copper have been correlated to cancer stage, recurrence and/or progression [12–17].

All together these findings suggest that copper might be a diagnostic/prognostic marker [10] and support the idea that the homeostatic machinery of this metal ion could be used as tumour-specific target. Since the 1965 [18], the copper-related tumour progression, has stimulated the development of copper-specific chelating ligands capable to inhibit many malignant processes. These membrane-permeating ligands, that also include diimines and related derivatives, form simple and mixed copper(II) complexes [19–21] and cause an increase of the copper concentration inside cells larger than that resulting from treatment with copper salts alone [22]. Such a behaviour is similar to that shown by metal ionophores which also deliver and release copper to cells [23]. Amongst others, proteasome inhibition [24], generation of reactive oxygen species (ROS) [25], and DNA damage [26] are the mechanisms invoked to account for the anticancer activity of copper complexes.

The family of metal complexes named casiopeinas, i.e. copper-based ternary complexes with diimines and ligands with O, N or O, O donor atoms [27–31], exhibits *in vitro* anticancer activities greater than that of cisplatin for human cell lines [32]. This class of compounds also displays promising *in vivo* activity, often at lower concentrations and with lesser side effects than cisplatin [33]. Recently, the different and not mutually exclusive mechanisms of action of these copper coordination compounds with diimines have been reviewed [34]. These compounds have been found to cause tumour cell death by apoptosis [35, 36], both in caspase dependent and independent mechanisms [27, 31, 36], as well as by autophagy [32]. The activities of these metal complexes seem to be linked to: *i)* generation of ROS [31, 37] with DNA oxidation and degradation [38, 39], with concomitant depletion of antioxidants such as glutathione (GSH) [40, 41]; *ii)* mitochondrial toxicity [28, 30, 42] and *iii)* DNA damage through direct interaction with metal complexes (the interaction may take place through intercalation, coordination of the metal to the negatively charged phosphate groups, insertion into the minor groove, partial substitution of some coordinating groups in DNA) [43–45].

**Scheme 1:**
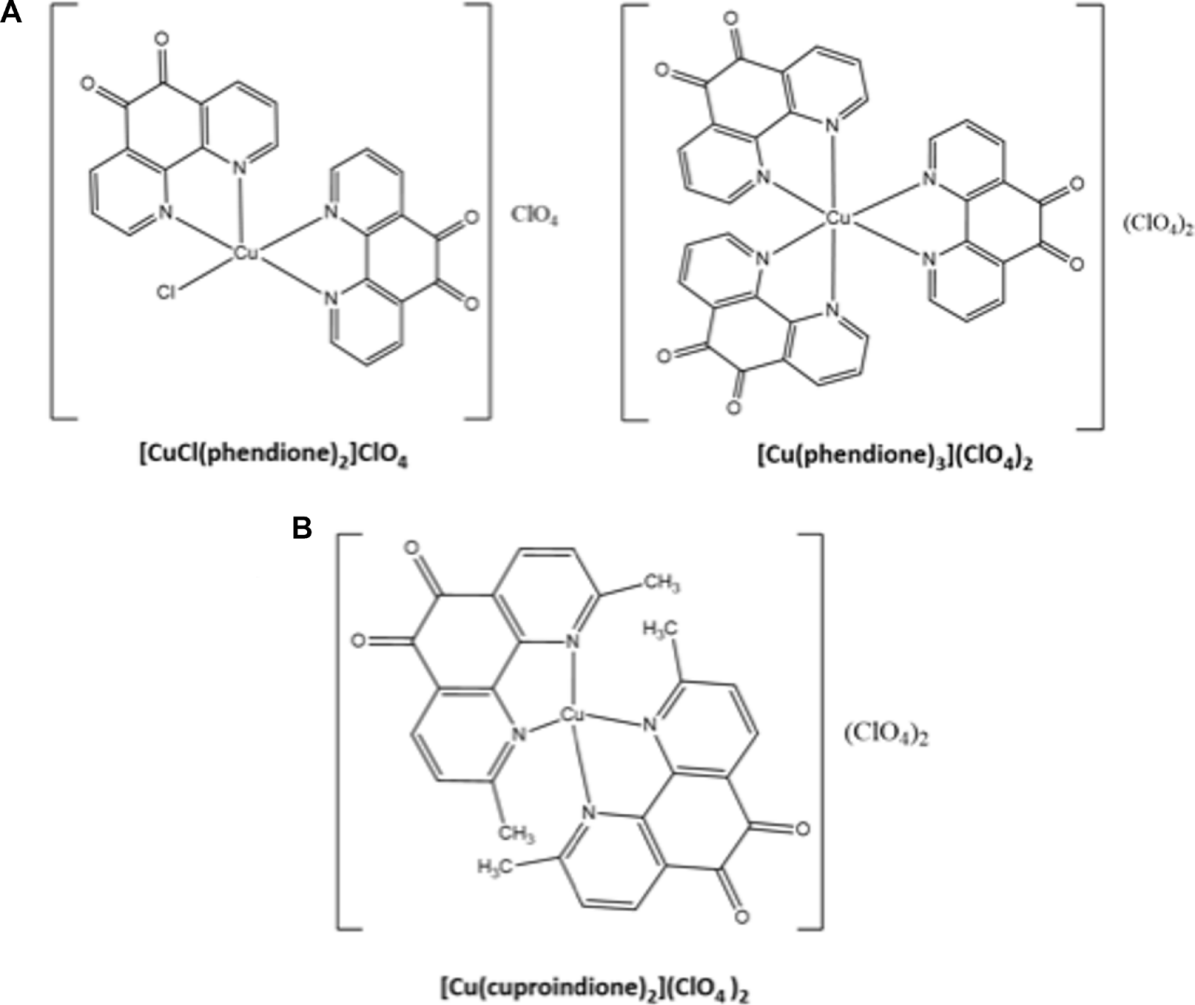
Structures of phendione (**A**) and cuproindione (**B**) compounds.

ROS, usually generated as side products [46] of the mitochondrial respiratory chain, when present at high levels may cause cell damage by regulating the expression of various apoptosis regulatory proteins [47]. Neoplastic cells possess higher ROS levels than normal cells [48]; consequently, a further increase of ROS may bring these levels to a lethal threshold [15], while resulting safer to normal cells [49]. Owing to its redox characteristics, copper may be involved in processes generating reactive oxygen species (such as the Fenton reaction and the Haber–Weiss reaction) [50], as well as in selective cytotoxicity against cancer cells. In both cases, copper efficiency depends on the properties of the ligands coordinated to the metal ion; for instance, substituents on the phenanthroline rings can affect differently the nuclease activity of the copper complexes [23, 51, 52]. Though the structure and biological properties of copper diimine complexes are still being investigated by various research groups [53–57], a distinctive feature of this class of ligands has attracted our attention: not only the copper complexes of 1,10-phenanthroline and 1,10-phenanthroline-5.6-dione, but also the bare ligands themselves are more cytotoxic than cisplatin *in vitro* [4, 58–61]. Surprisingly, the mechanism that lies behind such an activity has not received the attention it would have deserved. Being aware that 2,9-dimethyl-1,10-phenathroline and 1,10-phenanthroline form copper complexes having different structure and biological activities [62], here we report on and compare the cytotoxic activities of the copper(II) complexes with 1,10-phenanthroline-5,6-dione (hereafter named phendione) and its 2,9-dimethyl substituted analogue (hereafter named cuproindione) (Scheme 1) on the undifferentiated neuroblastoma cell line (SH-SY5Y). While metal complexes with phendione have already been investigated [63–69], cuproindione copper(II) complexes are reported here for the first time. The present investigation also shows how ROS production consequent to the cell culture treatment with the two 1,10-phenanthroline derivatives alters the metallostasis network (i.e., copper transporters and chaperones) [70] and the redox status of the cells.

## RESULTS AND DISCUSSION

### Synthesis and characterization of the compounds

The complex [Cu(cuproindione)_2_](ClO_4_)_2_·2H_2_O was prepared, isolated and characterized (see Material and Methods) by adapting the procedure used for the preparation of the complexes with phendione [71]. The infrared data highlight the differences between the phendione and the cuproindione copper(II) complexes. The spectrum of [Cu(cuproindione)_2_](ClO_4_)_2_·2H_2_O displays a strong band at 1699 cm^–1^ that is assigned to the *v*(C=O) stretching of coordinated cuproindione [71]. For free cuproindione this band falls at *ca* 1694 cm^-1^ (data not shown). The band at about 1590 cm^–1^ is attributed to *v*(C=C) of the aromatic ring [72]. The IR spectra of phendione are fairly similar to those obtained for cuproindione, and display smaller shifts for both the *v*(C=O) stretching and the *v*(C=O) stretching of the aromatic ring [73]. These bands are detected at *ca* 1700 cm^-1^ and 1685 cm^-1^ (for coordinated and free phendione, respectively) and at 1576 cm^-1^ for *v*(C=C) stretching.

**Figure 1:**
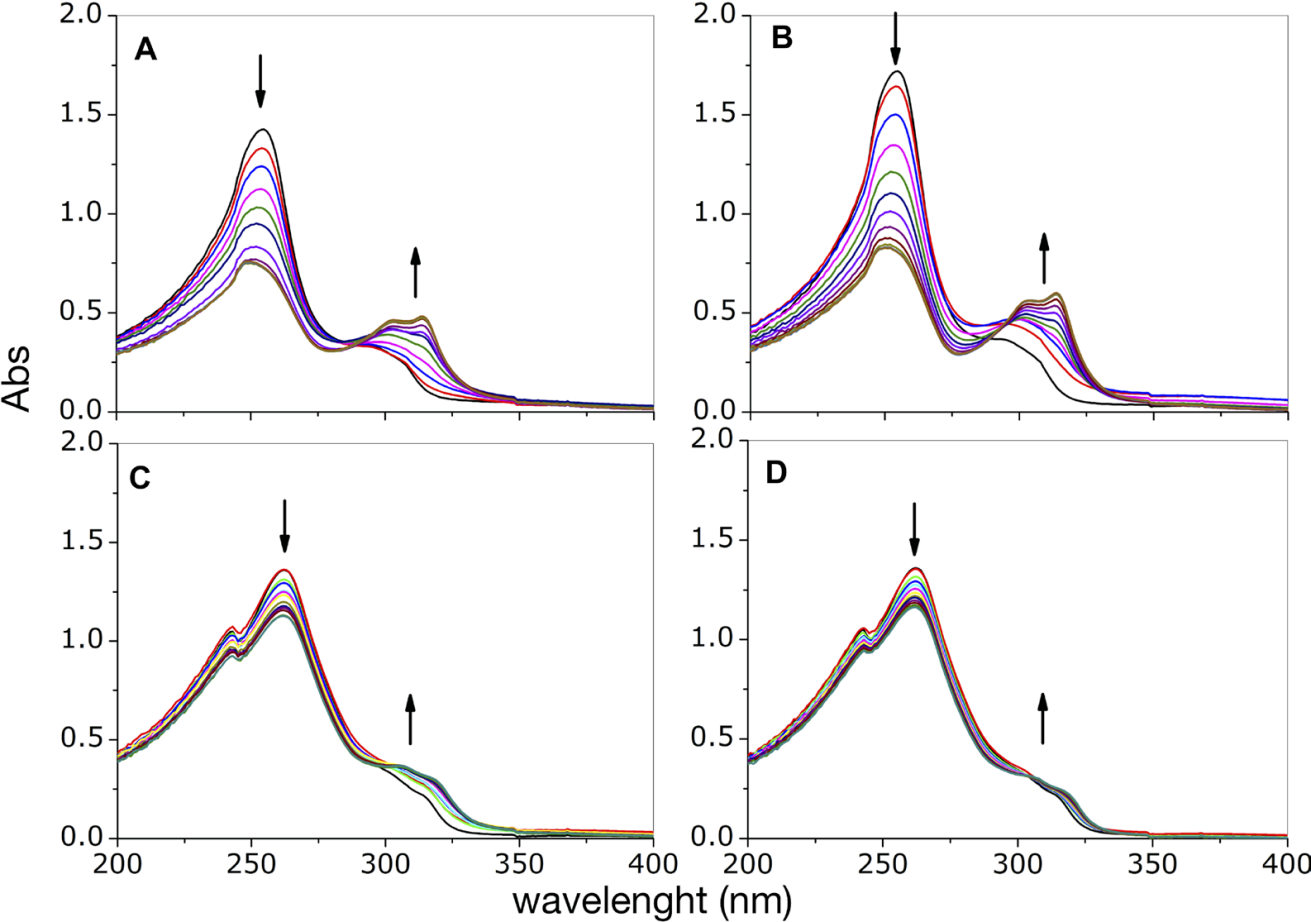
UV-visible curves of phendione (**A, B**) and cuproindione (**C, D**) treated with increasing equivalents (0.2- 1.2 range) of CuCl_2_ (A, C) or Cu(ClO_4_)_2_ (B, D).

In order to gain information on the complexes that form in solution, we followed the changes resulting from addition of copper(II) ion into a solution containing either phendione or cuproindione by UV-vis spectrophotometry (Figure 1). Phendione shows two main absorption bands, centred at 254 and 300 nm. The addition of increasing amounts of copper chloride (from 0.1 to 1.2 equiv.) results in a decrease of the intensity of the band at 254 nm and an increase of the band centred at 300 nm that is also accompanied by a bathochromic shift; The spectrum also shows a quasi-isosbestic point around 284 nm (Figure 1A). Following the addition of 0.5 equivalents of CuCl_2_, the bands centred at 254 and 300 nm remain nearly constant and increases, respectively. Furthermore, the quasi-isosbestic point is no longer observed and a better-defined absorption band is detected at 314 nm. Slightly different results were obtained for the analogous experiment performed using Cu(ClO_4_)_2_ (Figure 1B). Copper(II) perchlorate addition also causes a progressive reduction of the intensity of the band at 254 nm and an increase of the bands around 300 nm, (also accompanied by a bathochromic shift) as well as the appearance of an isosbestic point centred at 295 nm rather than at 284 nm, as observed for CuCl_2_. For cuproindione, the addition of CuCl_2_ (from 0.1 to 1.2 equiv.) causes a gradual decrease of the intensity of the bands at 243 and 262 nm and an increase of the shoulder at 309.5 nm (Figure 1C). There is a quasi-isosbestic point centred at 302 nm. There are no significant spectral changes after addition of 0.5 equivalents of CuCl_2_. A similar result was obtained when using Cu(ClO_4_)_2_ (Figure 1D).

**Figure 2:**
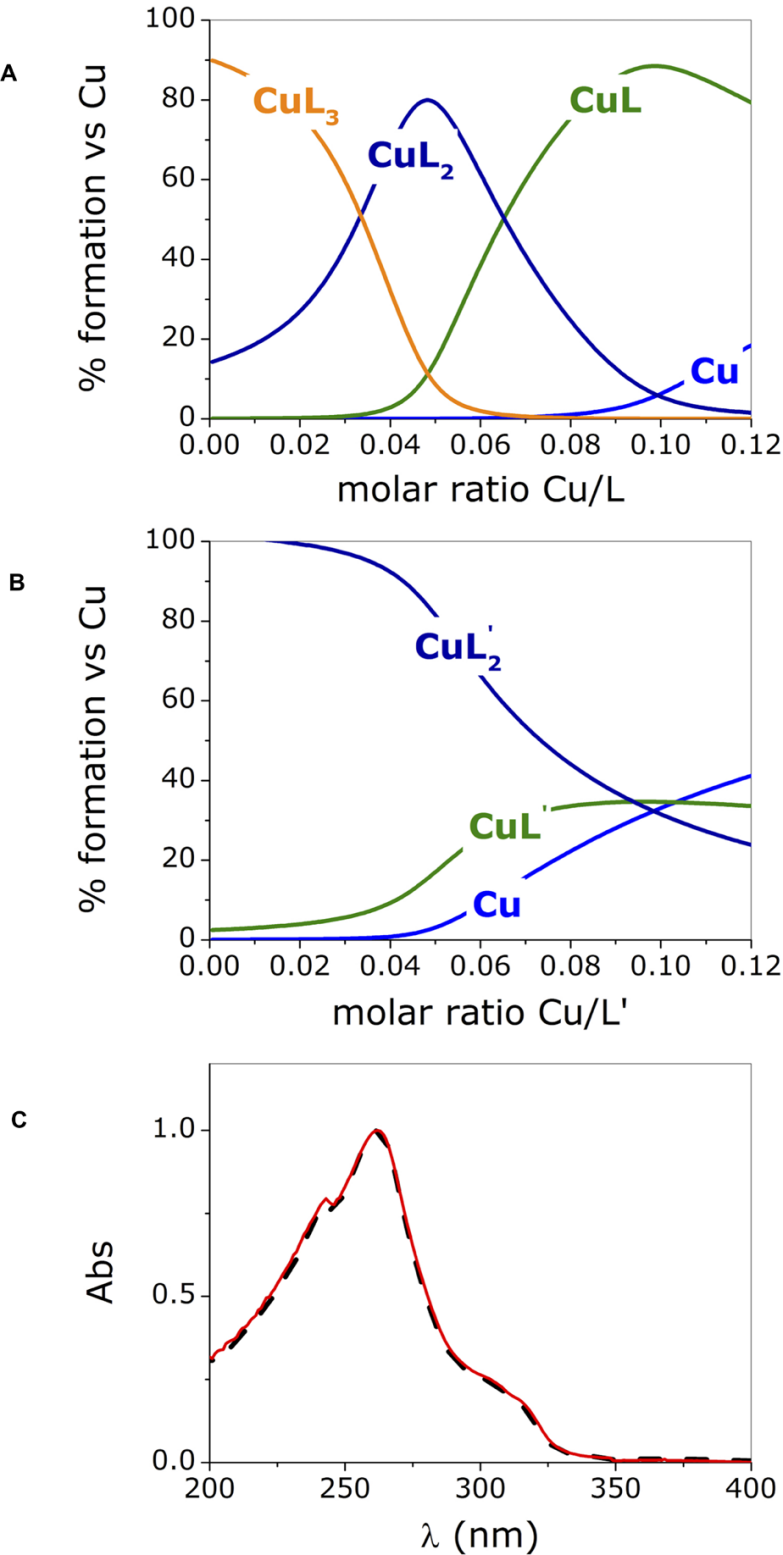
Species distribution computed for phendione (L) (**A**) and cuproindione (L’) (**B**) reproducing the conditions employed in the UV-visible experiments (charges are omitted for clarity); (**C**) UV-spectra of [Cu(cuproindione)_2_](ClO_4_)_2_·2H_2_O (solid red line) and a solution of cuproindione having a cuproindione/Cu^2+^ equal to 1:0.5 (dash black line). Titrate concentration= 6x10^-5^ M; titrant concentration= 1.8x10^-3^ M.

Figure 2 shows the species that form over the entire range of concentrations explored in the UV-vis titration experiments.

The distribution diagrams (Figure 2A, 2B) were obtained by using the equilibrium constants reported by Martell *et al.* [74] for 1,10-phenanthroline (phen) and 2,9-dimethyl-1,10-phenanthroline (2,9-phen), which both lack the carbonyl groups, under the assumption that the carbonyl groups will have similar effects on the two ligands. The assumption is based on a recently published paper showing that the stability constants of copper- phendione complexes is not significantly altered by the presence the carbonyl groups [61]. Figure 2A and 2B clearly show that the species formed in the titration with copper(II) of phendione and cuproindione, under identical experimental conditions are different. In the case of phendione, at least two species coexist (Figure 2A) over the entire range of molar ratios explored. As expected, in the initial part of the titration (i.e. in the presence of large excess of ligand) [Cu(phendione)_3_]^2+^ (CuL_3_) prevails over [Cu(phendione)_2_]^2+^ (CuL_2_); Beyond 0.4 equivalents of Cu^2+^, the percentage of the latter species rapidly decreases to give way to CuL_2_ that coexists with the mono-complex (CuL) for a large interval. At a 1:1 molar ratio, CuL is by far the dominant species accompanied by negligible amounts of CuL_2_ and free copper(II) ion. The UV-vis titrations (Figure 1A and 1B) reflect the simultaneous presence of overlapping species. The picture is less complex for cuproindione (Figure 2B), due to the formation of two species only, namely [Cu(cuproindione)_2_]^2+^ (CuL’_2_) and [Cu(cuproindione)]^2+^ (CuL’). However, as the 1:1 ratio is approached, the percentage of CuL’_2_ drops and roughly equivalent amounts (∼30%) of CuL’_2_, CuL’ and free copper(II) ion coexist in these conditions, owing to the smaller formation constants of cuproindione compared to phendione.

The UV-vis spectrum obtained by dissolving the synthesized complex, ([Cu(cuproindione)_2_] (ClO_4_)_2_·2H_2_O), mirrors the spectrum recorded for a solution with a Cu/L’ (cuproindione/Cu^2+^ 1:0.5) (Figure 2C). However, the spectrum of both solutions does not result from the presence of a single specie but rather reflects the complex picture resulting from the simultaneous formation of multiple species (Figure 2B). This clearly shows that a solid metal complex with a given stoichiometry does not necessarily survive in aqueous solution with the same stoichiometry, as more than one species can originate, whose concentration ratio depends on their affinity constants.

In order to ascertain the set of donor atoms involved in Cu^2+^ coordination, EPR measurements were also carried out. The metal to ligand ratios were chosen based on the calculated species distribution (Figure 2), to minimize the overlapping of different complex species. As to phendione, the EPR parameters for the CuL system (*g*_∥_ = 2.325 and *A*_∥_ = 158 × 10^–4^ cm^-1^) indicate the involvement of two nitrogen and two oxygen atoms in a tetragonal arrangement, thus confirming the presence of the [Cu(phendione)]^2+^ species. The parameters are similar to those reported for analogous complex species formed with 1,10-phenanthroline and its derivatives, in which the metal has the same coordination environment [44]. The EPR spectra obtained by increasing the ligand to metal ratio are characterized by a smaller g value (*g*_∥_ = 2.260) and a larger hyperfine coupling constant (*A*_∥_ = 175 × 10^–4^ cm^-1^), which indicate the substitution of oxygen by nitrogen atoms in the metal coordination environment. These EPR parameters suggest the presence of a CuN_4_ chromophore, that indicates the formation of a species, [Cu(phendione)_2_]^2+^, having nitrogen atoms in a nearly planar disposition. Noteworthy, these parameters closely resemble those reported for the bis-complex species formed by 1,10-phenanthroline [75].

As to cuproindione, the EPR parameters obtained for [Cu(cuproindione]^2+^ (*g*_∥_ =2.323 and *A*_∥_ =154 × 10^–4^ cm^-1^), are comparable to those obtained for the [Cu(phendione)]^2+^ species, pointing to the presence of a similar CuN_2_O_2_ chromophore. As the metal/ligand ratio increases, the EPR spectra reveal the simultaneous presence of different complex species. At 1:2.5 metal/ligand molar ratio, the parameters of the main species (*g*_∥_ = 2.293, *A*_∥_ = 162 × 10^–4^ cm^-1^) are analogous to those observed for the copper(II) complex species formed by 2,9-dimethyl-1,10-phenanthroline [44, 76]. Such parameters indicate a stronger ligand field compared to [Cu(cuproindione]^2+^, suggesting the presence of more nitrogen atoms around the metal ions and thus the formation of Cu(cuproindione)_2_]^2+^ having a tetragonal geometry more distorted than that of the analogous species formed by phendione.

### Cytotoxicity of phendione and cuproindione: the chelators transport copper inside the cell and induce cytotoxicity

Dose-response experiments were performed to compare the activity of cuproindione and phendione on an undifferentiated neuroblastoma cell line (SH-SY5Y). Cells were treated with each compound for 48 hrs exploring the 0.01–10 µM concentration range. The IC_50_ values of cell viability (MTT assay), obtained by a linear regression of the dose response curves of each compound are reported in Table 1.

**Table 1:** Cytotoxicity (as IC_50_ values, in 10^-6^ M) of phendione (L), cuproindione (L’), [CuCl(phendione)_2_]ClO_4_·3/2H_2_O (1), [Cu(phendione)_3_](ClO_4_)_2_×4H_2_O (2), [Cu(cuproindione)_2_] ClO_4_)_2_·2H_2_O (3) and cisplatin (4, [77])

L	L’	1	2	3	L+ BCS	L’+ BCS	4
1.5 ± 0.1	0.78 ± 0.09	0.41 ± 0.02	0.38 ± 0.03	0.39 ± 0.03	1.56 ± 0.02	4.0 ± 0.1	2

The two ligands (L and L’) show IC_50_ values lower than that reported for cisplatin, with cuproindione being more cytotoxic than phendione. Moreover, the copper(II) complexes exhibit cytotoxicity values five times greater than that of cisplatin, similarly to what previously reported for [Cu(phendione)_3_](ClO_4_)_2_ and [Ag(Phendione)_2_](ClO_4_), which induce a dose-dependent decrease of DNA synthesis [59]. Analogous results by Igdaloff *et al.* [58] for metal-free phendione inhibiting the growth of mouse-derived lymphoma cells, were related to the inhibition of DNA and RNA synthesis, as confirmed by Deegan *et al.* in DNA synthesis studies dealing with human kidney adenocarcinoma and human hepatocellular carcinoma cells [59]. However, other hypothesis have been raised and the anti-proliferative activity of phendione has been attributed to an unspecified cellular microenvironment [61].

Since metal salts are essential components of cell culture media where copper(II) concentration ranges from 0.94 µM to 1.9 µM [78], cells were also pre-treated for 3 hours with BCS (bathocuproine disulfonate), a copper chelator unable to cross the cell membrane [79].

**Figure 3:**
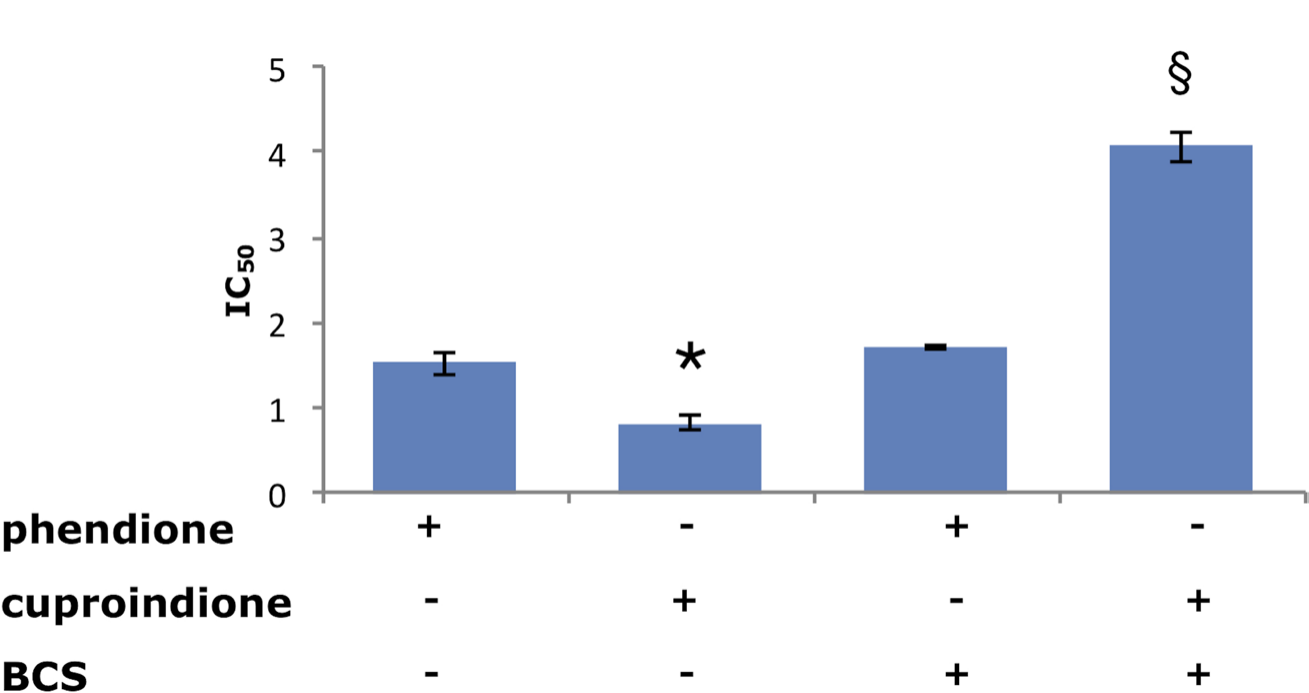
Viability of SH-SY5Y cells assessed by IC_50_ values (µM) following a 48-hrs treatment with phendione or cuproindione, either in the basal culture medium or after pre-incubation (3 hrs) with 50 µM BCS Values are expressed as mean ± SEM over at least three independent experiments. (^*^=*p* ≤ 0.05 level vs phendione, §=*p* ≤ 0.05 level vs cuproindione; One-way Anova).

Figure 3 shows that the ability of BCS to remove Cu^2+^ from the medium affects the anti-proliferative ability of the two ligands differently. Indeed, the toxicity of cuproindione decreases approximately by a factor of four under copper deprivation conditions, whereas the toxicity of phendione is unaffected by BCS. The large effect of BCS upon the toxicity of cuproindione can be explained by the very similar stability constants of copper(II) complexes with BCS and 2,9-phen, (used to mimic the copper(II) affinity of cuproindione). Thus, the large amount of BCS (not permeable to cell membrane) is likely responsible for sequestering Cu^2+^ outside the cell. By contrast, the negligible effect of BCS upon the toxicity of phendione can be explained by the different affinity constants of Cu^2+^ complexes with BCS and phen (used to simulate the copper(II) affinity of phendione). The affinity constant of the Cu^2+^ complex with phen is four orders of magnitude larger than that with BCS. Thus, BCS cannot compete with phendione for copper(II) coordination, notwithstanding its larger concentration. These findings highlight the role of Cu^2+^ present in the culture medium and may account for the different cytotoxicity of the two phenanthroline derivatives, whose activities can be attributed to their copper complexes. Although both ligands have similar copper chelating features, the distribution diagrams, computed under the assumption that the complexation characteristics of phen and 2,9-phen roughly parallel those of phendione and cuproindione, respectively, highlight the different percentages of complex species that form in the two cases (Supplementary Figure 1).

The distribution diagrams obtained by using the Cu^2+^ concentration reported for the medium by Huang *et al.* [78] show that for both ligands the mono- and bis-complex are the dominant species over the range of ligand concentrations investigated (0.01 µM - 10 µM) in the biological assays. At small ligand to metal (L/Cu or L’/Cu) ratios, CuL (or CuL’) is the main species; however, CuL reaches percentages as large as 90% (Supplementary Figure 1A) only in the case of phendione thanks to the higher affinity of phen for Cu^2+^ compared to 2,9-phen (Supplementary Figure 1A and 1E). As the L/Cu (or L’/Cu) ratio increases (Supplementary Figure 1B–1D and 1F–1H), CuL_2_ and CuL’_2_ override the formation of the CuL (or CuL’) species. CuL_3_ forms only in the case of phendione and in the presence of excess of ligand. However, even when the ratio is pushed up to reproduce roughly the conditions of the toxicity found in the MTT assay, CuL_2_ and CuL’_2_ are still the main species in both systems and reach roughly the same percentage (Supplementary Figure 1D and 1H). CuL_2_ stimulates hydroxyl radical formation from molecular oxygen by redox cycling and thus might promote oxidative stress [59]. The reaction pathway proceeds *via* the formation of a cuprous complex in solution ([Cu(phen)_2_]^+^), characterized by a non-intercalative binding of the tetrahedral metal complex in the DNA minor groove [80]. The [Cu(2,9-phen)2]+ stability constant (log β = 19.1) is significantly larger than that of [Cu(phen)2]+ (log β = 15.8) [81], due to the more favourable disposition of the methyl groups of 2,9 phen in the tetrahedral copper(I) bis-chelate complex.

The transcription inhibitor ability of [Cu(2,9-phen)_2_]^+^ contributes to its cytotoxicity on eukaryotic and prokaryotic cells [82]; altogether, these evidences can justify the copper-dependent different anti-proliferative activity of cuproindione and phendione (Table 1). The greater stability of the cuprous complex may explain, at least in part, the larger anti-proliferative effect of cuproindione with respect to phendione.

### Phendione and cuproindione induce nuclease activity, oxidative stress, and apoptosis and affect mitochondria by p53 activation

Several factors have been invoked to explain the different nuclease activity of the mono and bis-complexes of copper (II)/copper(I) with 1,10-phenanthroline and its derivatives [51, 62, 83–86]. These include: *a)* stoichiometry; *b)* metal complex geometry (planar *vs* tetrahedral) [61, 87, 88]; c) redox ability (phen *vs* 2,9-phen complex species) [62, 89, 90]; *d)* metal ion (Cu^+^ vs Cu^2+^) affinity for the ligand; e) phen and 2,9-phen complex species interaction with reductants within the cell (ternary complex formation with thiols by mono-complex species vs. thiol oxidation by bis-complex species) [91–94]; *f)* copper complex species interaction with DNA (intercalation *vs* minor groove binding) [31, 43–45].

Previous studies have focused on copper only, working in a cell-free environment and without differentiating between cupric and cuprous complexes, which have both different stability constants and redox potentials. This may account for some misleading reports on the cleavage efficiency of mono- and bis-complex of copper with phen and 2,9-phen [24, 95, 96].

The biochemical assays carried out in the present work may help unravelling some of the above still unresolved questions. To ascertain whether also in living cells a DNA damage could be one of the toxic effect of phenanthroline derivatives, cells were treated with the compounds under investigation and specific markers (e.g. Poly [ADP-ribose] polymerase 1 (PARP-1)) were analysed. PARP-1 is a well-known apoptotic marker and undergoes proteolytic cleavage by activated caspase 3 [97]. PARP-1 is very sensitive to cleavage as it controls genomic stability [98]. Supplementary Figure 2 shows the assays carried out by Western Blot (WB) analysis for PARP-1 expression (at metal-to-ligand ratios lower than 1, with ligand concentration lower than IC_50_) and for PARP-1 cleavage (at IC_50_ values of the two ligands where the cuprous bis-complexes are the main species). The expression of PARP-1 (Supplementary Figure 2), upon treatment with non- toxic concentrations of the ligands (until 0.1 µM), increases with respect to control and reaches similar values with both phendione and cuproindione complexes. Since the induction of PARP-1 expression may be indicative of an interaction between these copper-binding phenanthroline derivatives and DNA, with a consequent endonuclease cleavage [68], our results suggest that the species that forms under these conditions (i.e., CuL or CuL’) interacts with DNA by intercalation and cause a lengthening, stiffening, and unwinding of the helix [99] that is sensed by PARP-1. The lack of toxicity and oxidative cleavage may be ascribed to the formation of ternary complexes of copper(I)-phendione or cuproindione with thiols (likely glutathione), as observed in cell-free experiments employing copper-phendione complexes that showed a delayed oxidative nuclease effect [96]. Following treatment with IC_50_ concentrations of ligands, the cleaved PARP-1/full PARP ratios (Supplementary Figure 2B), increases by about 120% and 150% (compared to control) for phendione and cuproindione, respectively. For both compounds, the pre-treatment with BCS nullifies the enhanced PARP-1 cleavage compared to the control. We also tested the PARP-1 24 kDa cleaved fragment and obtained roughly the same result (data not shown).

The different oxidative nuclease efficiency of phendione and cuproindione may relate both to the different amount of cuprous species (resulting from the larger affinity constant value of the metal-bis ligand complex species of 2,9-phen compared to phen, log β = 19.1 *vs* 15.8) and to the higher redox potential of the cupric complexes with 2,9-phen relative to phen (+594 *vs* +174 mV) [81]. These two parameters affect the oxidative efficiency of the two systems toward GSH, the most relevant reductant in the cytosol (GSH/GSSG redox potential < −300 mV [100]).

**Figure 4:**
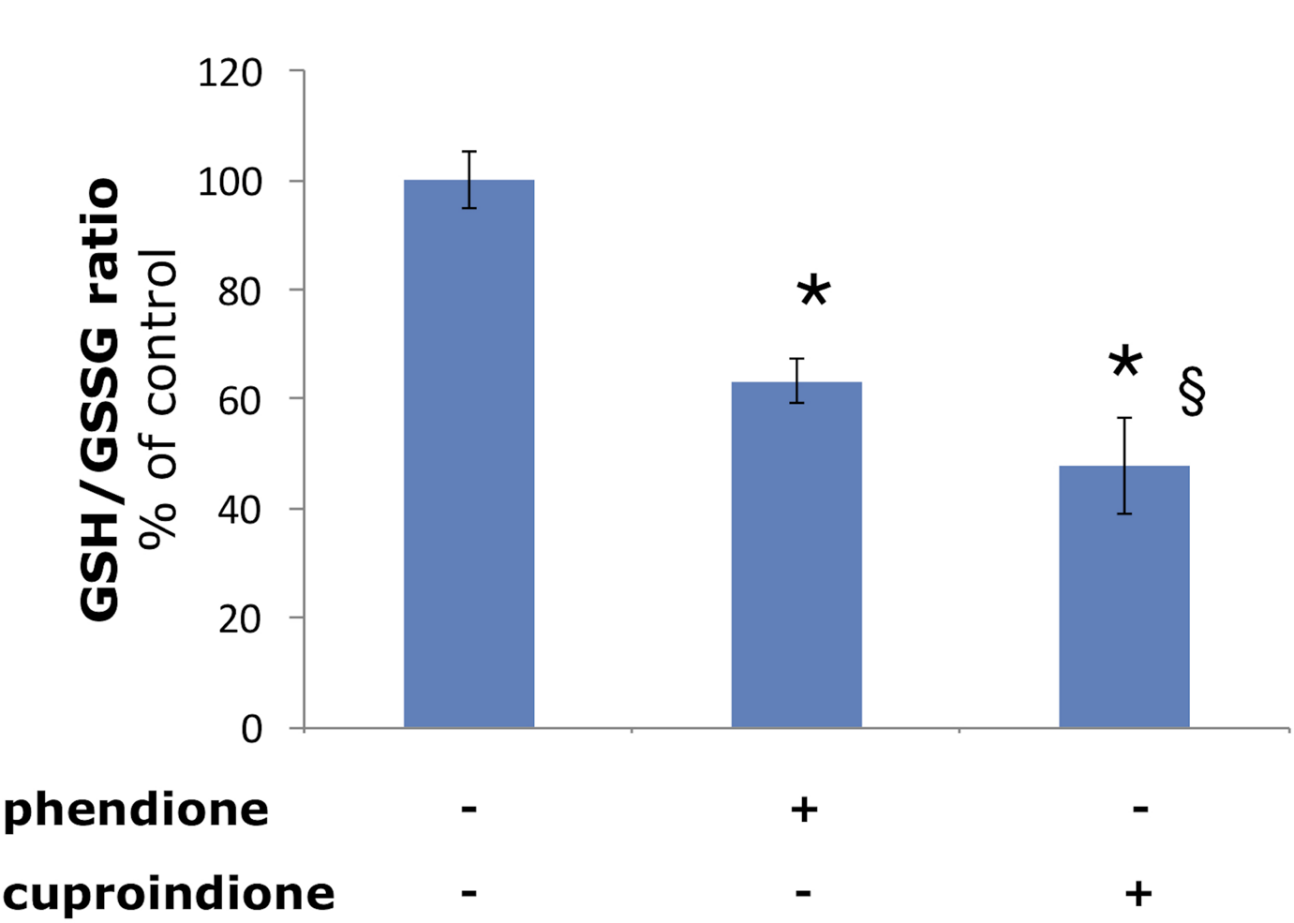
Effect of phendione or cuproindione treatment on GSH redox state in SH-SY5Y cells treated with a phendione or cuproindione 3 × IC_50_ concentration for 90 minutes Results are expressed as mean ± SEM from triplicate experiments and normalized with respect to the control (untreated) cells. (^*^p ≤ 0.05 level vs control; ^§^*p* ≤ 0.05 level vs phendione; One-way Anova).

In agreement with such hypothesis, we found a decrease of the GSH/GSSG ratio in the cell that is more pronounced for cuproindione than for phendione (Figure 4). This supports the effective role played by the methyl groups of cuproindione and evidences the prevailing role of Cu^+^ complexes over Cu^2+^ species. Such a trend has also been reported for the analogous complexes lacking the carbonyl groups [24].

The mechanism of apoptosis is a multifaceted process under the control of different pathways and cell life check control. Since copper levels are significantly elevated in a number of malignancies, cancer cells would be more subject to redox cycling between copper species and thus generate larger amounts of ROS, responsible for DNA breakage [101]. In addition to DNA damage and specific cell death signalling, mitochondrial activity disturbances may be sensed by cells as critical steps to the decision for suicide or survival.

Redox cycling between Cu^2+^ and Cu^+^ can be efficiently mediated by different compounds exerting cancer cell toxicity owing to a copper related mechanism [102]. The same mechanism might be responsible for mitochondria destabilization and apoptosis activation. Mitochondrial destabilization can easily be followed by monitoring the membrane potential (Δψ_m_), that is rapidly affected by many toxic compounds and oxidative stress as well as by excitotoxic conditions [103].

**Figure 5:**
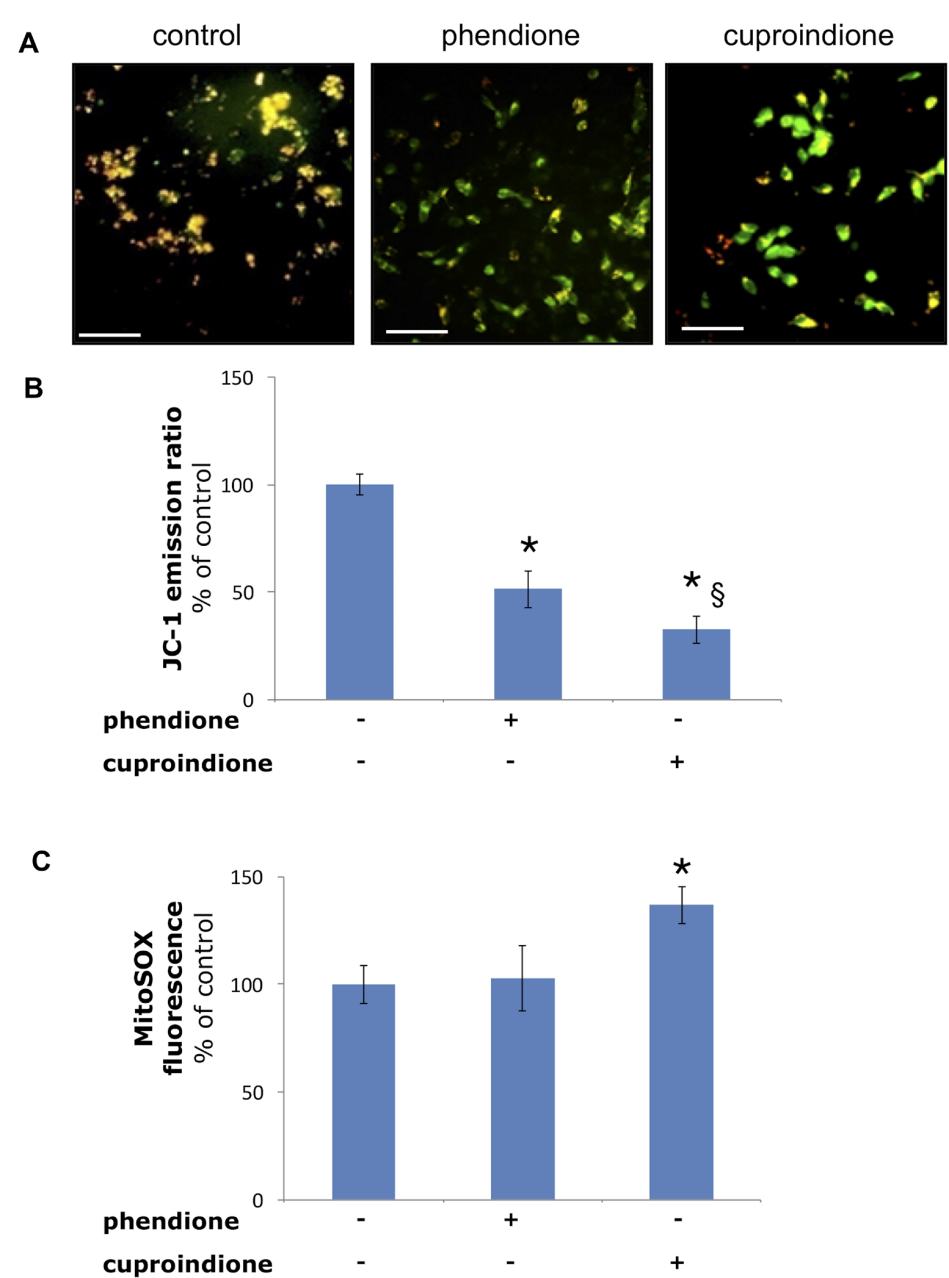
Mitochondria polarization of the cells treated with a phendione or cuproindione 3 × IC_50_ concentration for 90 minutes (**A**) fluorescence micrographs; (**B**) calculated ratios of JC-1 fluorescence emission at 595/530. Mitochondrial O2•− production of the cells treated with a phendione or cuproindione 3 × IC_50_ concentration for 30 minutes: (**C**) MitoSOX emission (ex 510, em 580). Results are expressed as mean ± SEM over three independent experiments with eight replicas for each condition (^*^= *p* ≤ 0.05 level vs control, ^§^ = *p* ≤ 0.05 level vs phendione; One-way Anova). Scale bar = 50 µm.

Figure 5 shows the changes of the mitochondrial membrane potential as well as mitochondrial ROS production following treatment with phendione or cuproindione. The mitochondrial membrane potential was measured through a specific mitochondrial fluorophore, JC-1, which undergoes a fluorescence emission change if depolarization occurs. Figure 5A, 5B show that JC-1 accumulates in the mitochondria of healthy cells under the form of aggregates (red-orange emission), whereas it remains in the cytoplasm in its monomeric form (green fluorescence) in cells treated for 90 min with phendione or cuproindione, as a result of the collapse of Δψ_m_. Noteworthy, the amount of O_2_^•−^ detected in treated cells (30 min) by monitoring MitoSOX emission is significantly higher for cuproindione (Figure 5C). This suggests a different efficiency of the two compounds, with cuproindione having a stronger effect on the permeability of the mitochondrial membrane. The alteration of Δψ_m_ through the generation of ROS can both provide evidence of the disruption of the outer mitochondrial membrane [104] and indicate an important pathway of induced apoptosis, as that attributable to p53 [105].

**Figure 6:**
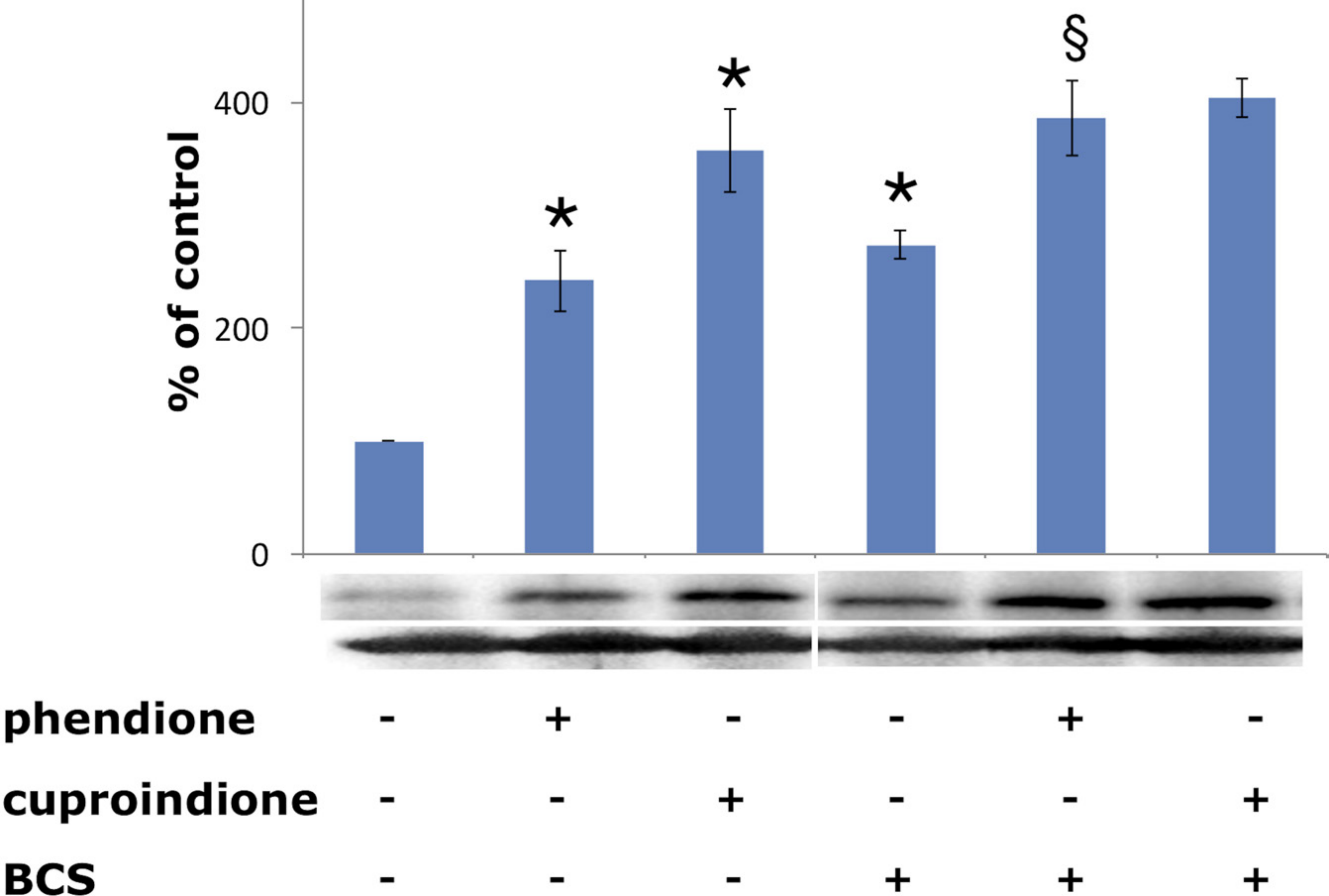
Expression of p53 in SH-SY5Y cells after a 48-hr treatment with phendione and cuproindione IC_50_ concentration in the absence and in the presence of 50 µM BCS Results are the mean ± SEM over at least three independent experiments. (^*^p ≤ 0.05 level vs control, ^§^*p* ≤ 0.05 level vs phendione; One-way Anova).

The tumour suppressor protein p53 is a zinc-binding transcription factor [106], often inactivated in cancer cells [107]. DNA damage causes p53 accumulation in the nucleus, binding to a DNA specific sequence, and the transactivation of several target genes [108] involved in cell cycle [109] and apoptosis [110]. DNA strand breaks, induced by oxidative stress [111], is one of the most potent signals leading to p53 induction. Consistently with their diverse oxidative nuclease ability, phendione and cuproindione up regulate differently p53 expression (Figure 6). Noteworthy, once again copper relevance emerges because pre-treatment with BCS only increases the expression of p53. The p53 protein itself is redox-sensitive and contains several cysteines located within the DNA-binding domain [112]. Three of these cysteines and a histidine are involved in the tetrahedral coordination to zinc, leading to a protein arrangement that interacts with the minor groove of target DNA. Several other cysteines are located in the regions that bind within the major groove of target DNA [113]. A murine *in vitro* assay showed that physiological concentrations of copper(II) ion perturb the conformation of wild-type p53 and inhibit its sequence-specific DNA-binding, while BCS protects against the effect of Cu^2+^ and prevents the metal ion cellular uptake [114]. The effects of ionophores (e.g. pyrrolidinedithiocarbamate, PDTC) and chelating ligands (e.g. phen) on p53 activity further support the above results and highlight the differences between copper(II) ionophores and chelating ligands [115]. PDTC increases intracellular copper concentration [57, 116], affecting the p53 pathway. The ionophore inhibits the nuclear translocation of p53, induces a “wild-type” to “mutant” transformation, and down-regulates the DNA-binding activity of p53. These multiple effects result from an increase in the oxidation state of p53 cysteine thiols. In turn, the increased oxidation may result from the capacity of PDTC to bind extracellular copper and release it within the cell [117].

Like phendione and cuproindione, phen also favours copper internalization in the cell; however, in the presence of phen, the process takes place through a complex species that upregulates p53 DNA-binding activity *via* a DNA damage-dependent pathway [57]. Though having similar effects on intracellular copper levels, PDTC and phen have opposite effects on the DNA-binding activity of p53. Furthermore, our findings on phenathroline derivatives further indicate that the effects of phen and PDTC cannot solely result from their ability to increase intracellular copper levels but also depend upon the redox activity of the metal species formed by the two ligands within the cell.

A marked connection between p53 and B-cell lymphoma-2 (Bcl-2) protein family has been reported [118]. The mitochondrial apoptotic pathway is strictly regulated by selective interactions between anti-apoptotic and pro-apoptotic proteins belonging to the Bcl-2 family [119]. This family regulates the intrinsic apoptotic pathway by controlling the mitochondrial outer membrane integrity [120], preventing the p53-induced Δψ_m_ alteration [105], and halting apoptosis through an anti-oxidative effect [121, 122]. On the other hand, Bcl-2 may contribute to inhibit caspase-dependent cell death by modulating the activity of the copper-dependent cytochrome c oxidase, the terminal complex of mitochondrial respiratory chain [123]; consequently, copper homeostasis derangements [124] or the Bcl-2 down-regulation, represent critical mitochondrial targets in fighting tumour cells. Bax, one of pro-apoptotic Bcl-2 family protein, can be activated by p53 [125] during apoptosis with the translocation of Bax from the cytosol to mitochondria [126]. Both *in vitro* [127] and *in vivo* [128] assays indicate that the pro-apoptotic effects of Bax may be elicited through an intrinsic pore-forming activity [129] accompanied by the release of cytochrome *c* [130].

Supplementary Figure 3 shows that short-time treatments (90 min) with phendione and cuproindione induce Bax translocation to the mitochondrial membrane (Supplementary Figure 3A) as well as cytochrome C release (Supplementary Figure 3B). Cuproindione is more effective than phendione, consistently with p53 and JC-1 findings. By contrast, long-time treatments (48 hrs) with the two compounds down regulate Bcl-2 expression (Supplementary Figure 3C). The pre-treatment of the medium with BCS nullifies the effect of cuproindione that forms weaker complexes than phendione, highlighting the relevance of the larger stability of copper-phendione complexes in the competition with BCS.

The ratio between Bcl-2 and Bax could determine the fate of cells undergoing apoptosis. The tight control of apoptosis is of critical importance for cancer cells to overcome their highly stressing condition and to counteract the constitutive expression of pro-apoptotic proteins. Noteworthy, the ratio between Bcl-2 and Bax has paved the way to target cell death in cancer and monitor the responses to therapies [131]. The effects observed following cell cultures treatments may well fit in this pro-apoptotic anticancer strategy, and highlight the participation of the copper homeostatic machinery in the apoptotic control of cell life, thereby indicating a new possible way to induce tumour cells to death.

### Oxidative stress affects metallostasis

Cellular copper homeostasis is ensured by some copper binding proteins [132] that control concentrations, binding interactions and location of single metal species that determine copper metallome [70, 133]. Prominent modulators of copper homeostasis are: *i)* import (high-affinity copper transporter 1, CTR1 [134]) and export (ATPase) Cu^+^ transporters across membranes; *ii)* trafficking components, including small molecules (GSH) and chaperones (CCS, Atox1, COX17/Sco1-Sco2, COX17) that escort Cu^+^ inside cell [135]; *iii)* insertase agents, CCS and Sco1/Sco2 (that insert Cu^+^ into apo-SOD1 and cytochrome c oxidase, respectively), Atox1 (that transfers Cu^+^ to ATP7A and ATP7B); *iv)* storage molecules (metallothionines); and *v)* metal transcription factors (Atox1, p53, MTF-1, Sp1). These metallostasis [136] regulators can partially remedy copper ion dyshomeostasis, that is responsible for a broad range of human diseases, including tumours [137, 138].

**Figure 7:**
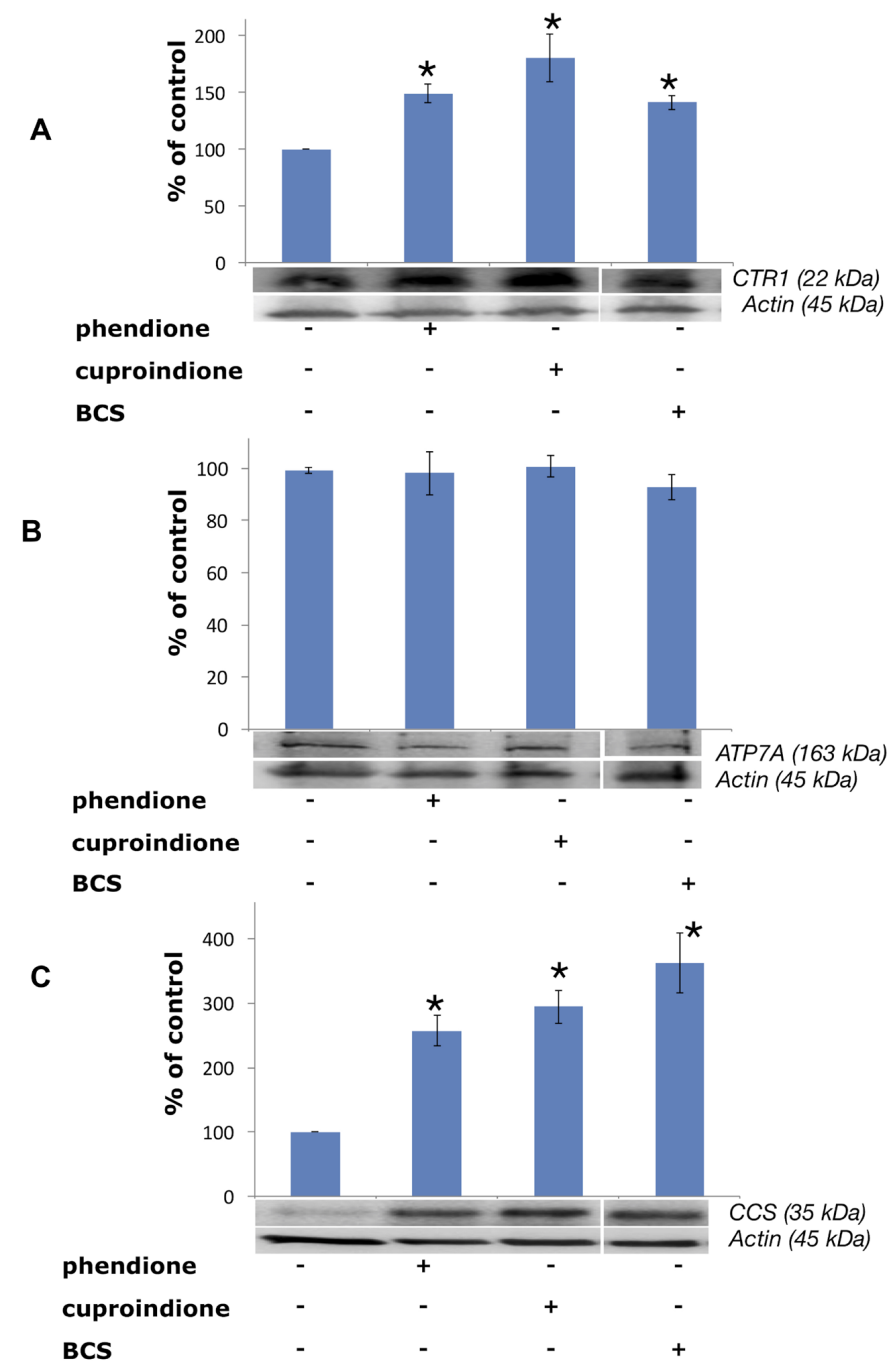
Expression of CTR1 (**A**), ATP7A (**B**) and CCS (**C**) in SH-SY5Y cells after treatment with a phendione (IC_50_ concentration), cuproindione (IC_50_ concentration) and BCS (50 µM) for 48 hrs. Results are expressed as mean ± SEM over at least three independent experiments. (^*^p ≤ 0.05 level with respect to control, One-way Anova).

Protein expression experiments were performed to evaluate how copper bound to phendione and cuproindione affects the copper homeostasis network of an undifferentiated neuroblastoma cell line (Figure 7). Figure 7A shows a significant CTR1 up-regulation in cells treated with BCS that sequestrates copper present in the medium and blocks the metal ion outside the cell. Like BCS, phendione and cuproindione also bind copper and markedly increase the expression of the protein devoted to the transferring of the metal ion inside the cell.

Noteworthy, CTR1 levels depend upon both the transporter localization and abundance [139]. The plasma membrane is the main location designed to respond to copper cellular demand, while the influx protein is mainly located in the membrane of intracellular vesicles in the presence of elevated exogenous copper [139, 140]. The strong binding of the metal ion to both ligands may account for the observed up-regulation.

Treating cells with membrane-impermeable copper chelators, including ATTM (ammonium tetrathiomolybdate), D-P (D-penicillamine) and BCS causes CTR1 up-regulation [141]. Conversely, these copper chelators do not alter the expression density and pattern of copper efflux ATPases in the same cell lines [142]. In line with such findings, our results show that the expression of ATP7A does not change when neuroblastoma cells are treated with BCS (Figure 7B). Similarly, the membrane permeable ligands reported here do not perturb the expression of ATPA7. The intracellular location of ATPases is controlled by cytoplasm copper concentration; in cultured cells exposed to physiological copper concentrations both ATPases are located in the *trans* Golgi network, to supply the metal ion to secreted cuproenzymes. In the presence of large copper concentrations, ATPases re-localise to either the plasma membrane (ATP7A) [143] or a vesicular compartment (ATP7B) [144].

The resulting metal efflux and compartmentalization, together with ATPases overexpression, have been associated with drug resistance in different tumors [145].

CCS is a 70 kDA Cu^+^ protein,^203^ that is required for the conversion of apo-SOD to holo-SOD, that, in turn, involves the conversion of specific thiols to disulphide [146], process that takes place in the presence of an oxygen-activated enzyme [147]. Figure 7C shows that CCS expression is upregulated by BCS as well as by the two ligands investigated in the present work, i.e., the chaperone behaves as if the cytosol were depleted of metal ion.

To ensure chaperone speciation and redox homeostasis, copper is transferred from a given protein to a different protein having a higher affinity [148], while the chaperone redox status is preserved by GSH (the most abundant antioxidant) that, together with its partners, is believed to maintain the redox potential in tissues, cells and individual compartments [100, 149].

The thiols of cysteines characterize the Cu^+^ binding to all components involved in metallostasis; in addition, oxidative stress, due to increased levels of Cu^+^ released within the cell by copper salts, can induce disulphide bond formation [150].

As a result, GSH is transformed into GSSG, while copper chaperone integrity is lost thus perturbing redox and chaperon speciation homeostasis [151].

Atox1 is another chaperon protein with specific properties [152]. It is a 68-amino acid protein where two of its three thiols bind Cu^+^; it also functions as a copper-dependent transcription factor [153] that mediates copper-induced cell proliferation. Atox1-deficient cells accumulate high levels of intracellular copper; metabolic studies indicate that this deficit results from an impaired cellular copper efflux [154].

**Figure 8:**
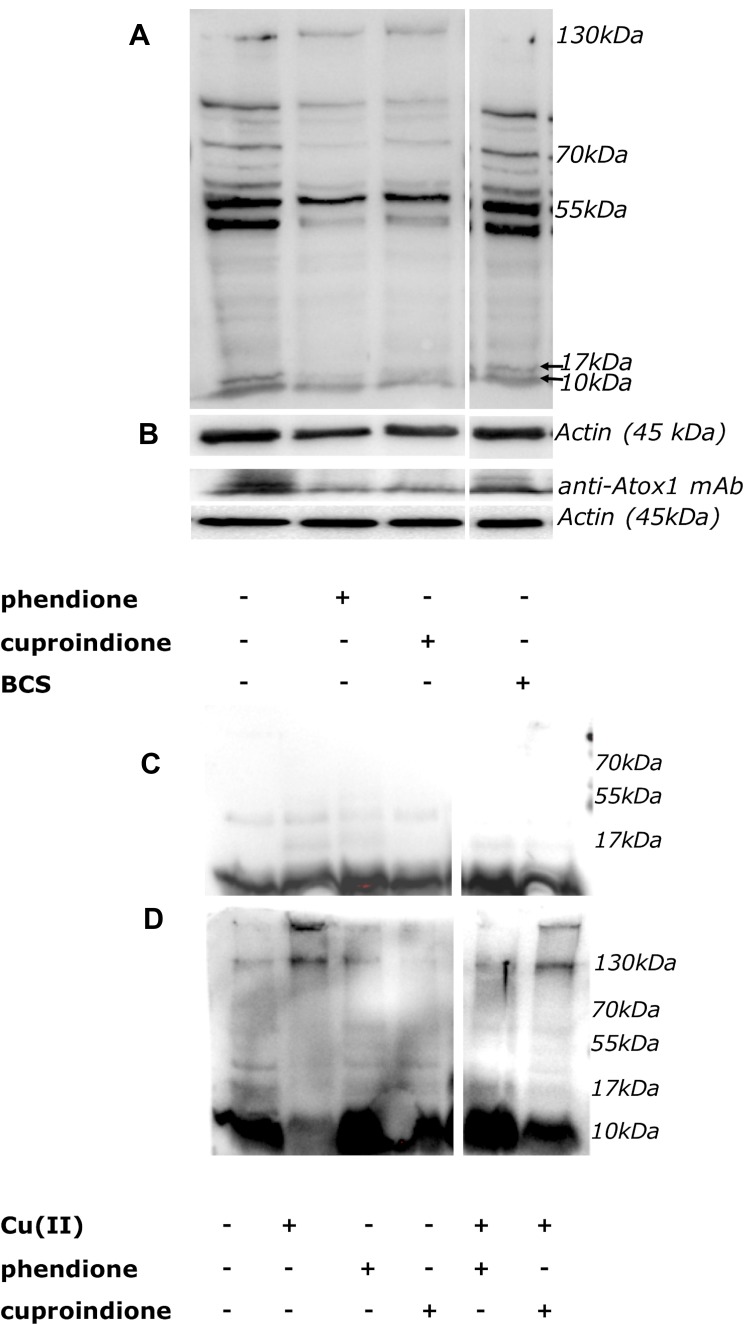
(**A, B**): Expression of Atox1 detected by polyclonal (A) or monoclonal (B) antibody in SH-SY5Y cells after 48 hrs of treatment. From the left to the right: control cells, cells treated with IC_50_ concentration of phendione or cuproindione or 50 µM BCS. (C, D): *In vitro* incubation of the Atox1 purified protein in the absence (**C**) or presence of H_2_O_2_ (**D**) for 20 hrs. From the left to the right: Atox1; Atox1 + Cu(II) (ratio 1:1); Atox1 + phendione (ratio 1:1); Atox1 + cuproindione (ratio 1:1); Atox1 + phendione + Cu(II) (ratio 1:1:1); Atox1 + cuproindione + Cu(II) (ratio 1:1:1).

Western Blot analyses of Atox1 by polyclonal or monoclonal antibodies are shown in Figure 8. Following a 48 hrs treatment with either phendione or cuproindione, oligomeric Atox 1 forms (i.e., the group of bands at higher MW, Figure 8A) increased and this was paralleled by a decrease of monomeric Atox1 (at a MW of approximately 8 kDa, Figure 8B). The *in vitro* incubation of recombinant Atox1 samples with copper only (Figure 8C) and with copper/H_2_O_2_ (Figure 8D) for 20 hrs results in the formation of dimers/tetramers and higher molecular weight species, respectively.

Atox1 is mainly present in a reduced form that is ensured by the markedly lower GSH steady-state redox potential [148]. The redox potential of GSH is mainly maintained by NADPH via GSH reductase [155]. In addition, NADPH in association with thioredoxin reductases reduces the oxidized thioredoxin (Trx-S-S) to its active dithiolic form [156] that is required under low GSH conditions [157]. Our experiments show that phendione and cuproindione copper complexes markedly decrease GSH levels (Supplementary Figure 4). Noteworthy, this is consistent with some reports showing that both free phendione and its complexes with some transition metal ions exhibit electro catalytic oxidations [66, 67]. All together these results justify the different redox forms of Atox1 found by WB assays. We might further hypothesize that under the pro-oxidant effect due to phenantroline treatments, ATOX1 oligomerization could affect its own function as copper efflux agent.

Evidence indicates that an increase of p53 due to oxidative stress caused by platinum compound results in the accumulation of nuclear copper in colorectal cells [158]. More recently, it has been demonstrated that in the same colorectal cell line treated with platinum compound [159], p53 can influence nuclear copper transport by affecting the regulation of Atox1 expression.

### Phendione and cuproindione induced oxidative stress affects the metallostasis network in mitochondria

Cellular copper homeostasis is accomplished by a highly complex and interconnected network of molecular interactions that balance: *i)* metal cytosol and sub-cellular uptake, *ii)* trafficking, *iii)* storage, *iv)* speciation and *v)* signaling [160].

**Figure 9:**
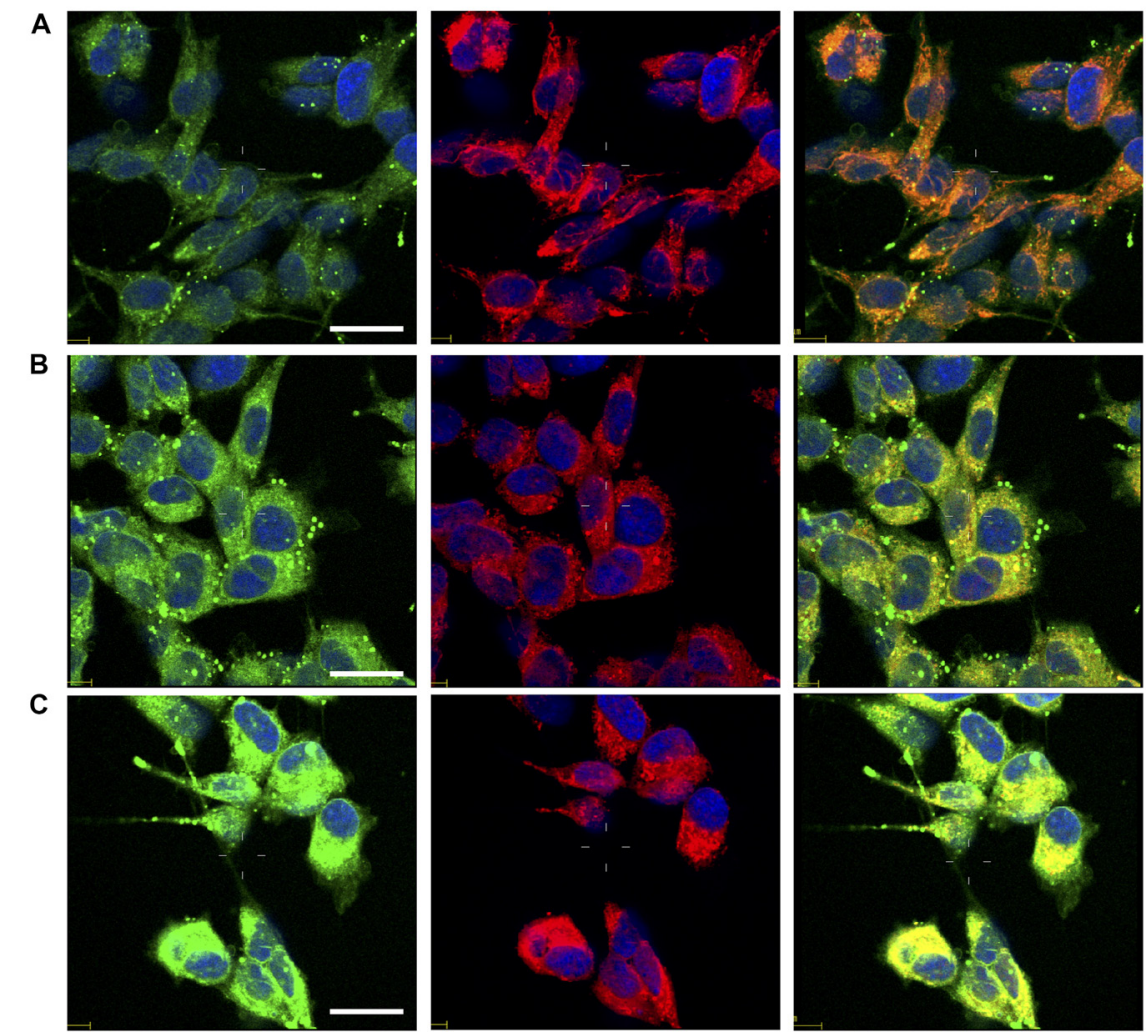
Confocal micrographs of untreated cells (**A**) and cells treated for 90 min with 4.4 mM phendione (**B**) or 2.2 mM cuproindione (**C**). After treatments cells were stained with copper sensor CS1 (green), Mitotracker Deep Red (red) and Hoechst33342 (blue). From the left to the right, merged channels for: copper + nuclei; mitochondria + nuclei; copper + mitochondria + nuclei. Scale bar= 30 µm.

In order to test whether the investigated compounds increase also nuclear copper in addition to p53, we monitored the subcellular changes of copper levels by laser scanning confocal microscopy (LSM) using CS1 [161, 162], a cell permeable chemosensor that specifically discriminates monovalent copper. Copper sub-cellular localization is illustrated in Figure 9. Compared to untreated control cells (Figure 9A), the cells treated with phendione (Figure 9B) and cuproindione (Figure 9C) show a cytosolic increased green fluorescence, consistent with Cu^+^ uptake. The presence of bright spots is likely due to the aggregation of the lipophilic CS1 probe in the aqueous intracellular environment [163]. The image analyses and the detection of CS1 emission at sub-cellular resolution (Supplementary Figure 5), indicate that the overall Cu^+^ content in cells treated with either phendione (Supplementary Figure 5B) or cuproindione (Supplementary Figure 5C) statistically increases both in the nuclei and in the mitochondria with respect to untreated control cells (Supplementary Figure 5A). We may conclude that the two compounds investigated cause Cu^+^ translocation into nuclear and mitochondrial compartments; in this context, cuproindione is more effective than phendione.

Mitochondria participate in a number of processes critical to cellular homeostasis, including the homeostatic maintenance of many metal ions like copper [164, 165] that is involved in the metal-assembly pathways of cytochrome *c* oxidase (CCO) and superoxide dismutase-1 (SOD1), the only two copper enzymes present within the mitochondria. Alterations of the metallostasis network in the cytosol, that drives the mitochondria enzyme metallation, causes fatal diseases characterized by CCO deficiency [166, 167]. Though the incorporation of metal ion in both SOD1 and CCO relies on the cysteine thiol redox status of the metallochaperones, the mitochondrial assembly of the copper sites in CCO involves a series of accessory proteins, including (but not limited to) COX17, Sco1 and Sco2; by contrast SOD1 needs CCS only. Sco1, Sco2 and COA6 [168], form a metallochaperone set to deliver and insert copper into the CuA site of CCO. This metallation process requires a dedicated upstream copper donor within the intermembrane space (IMS) of mitochondria; COX17 [169], that was previously hypothesized to shuttle copper between the cytosol and mitochondria based on its dual localization [170], is able to fulfil this role [171].

Although there are reports that have contributed to understand how Cu^+^ is trafficked to and within mitochondria [172], the IMS copper translocation and transfer to COX17 is still to unveil [173]; recently, a new mitochondrial copper transporter required for cytochrome c oxidase biogenesis has been identified [174]. There is no doubt, however, that COX17 transfers copper to both Sco1 and Sco2 [175]. This 69-residue cysteine-rich protein, containing three pairs of cysteines, has three oxidation states, namely an oxidized Cox17_3S–S_ with three disulphide bonds, a reduced Cox170_S–S_ with no disulphide bonds, and an intermediate state Cox17_2S–S_ with two disulphide bonds. Cox17_2S–S_ transfers Cu(I) and two electrons to the oxidized form of apo-Sco1 (containing a disulphide bond), but only to reduced Sco2 [176].

Copper is delivered to the CuA site only by Sco1. Sco2 acts as an oxidoreductase maintaining the cysteines of CuA under reduced conditions, thereby aiding copper delivery by Sco1 [177]. According to an alternative hypothesis, a ternary complex (Sco2-CCO-Sco1) would form and then Sco2 would sequentially transfer copper to CCO; Sco1 would then do the same process [178]. Following copper transfer, Sco2 acts as an oxidoreductase regulating the redox status of the cysteines in (oxidized) Sco1. This favours copper insertion into Sco1 by COX17 and activates the next cycle.

**Figure 10:**
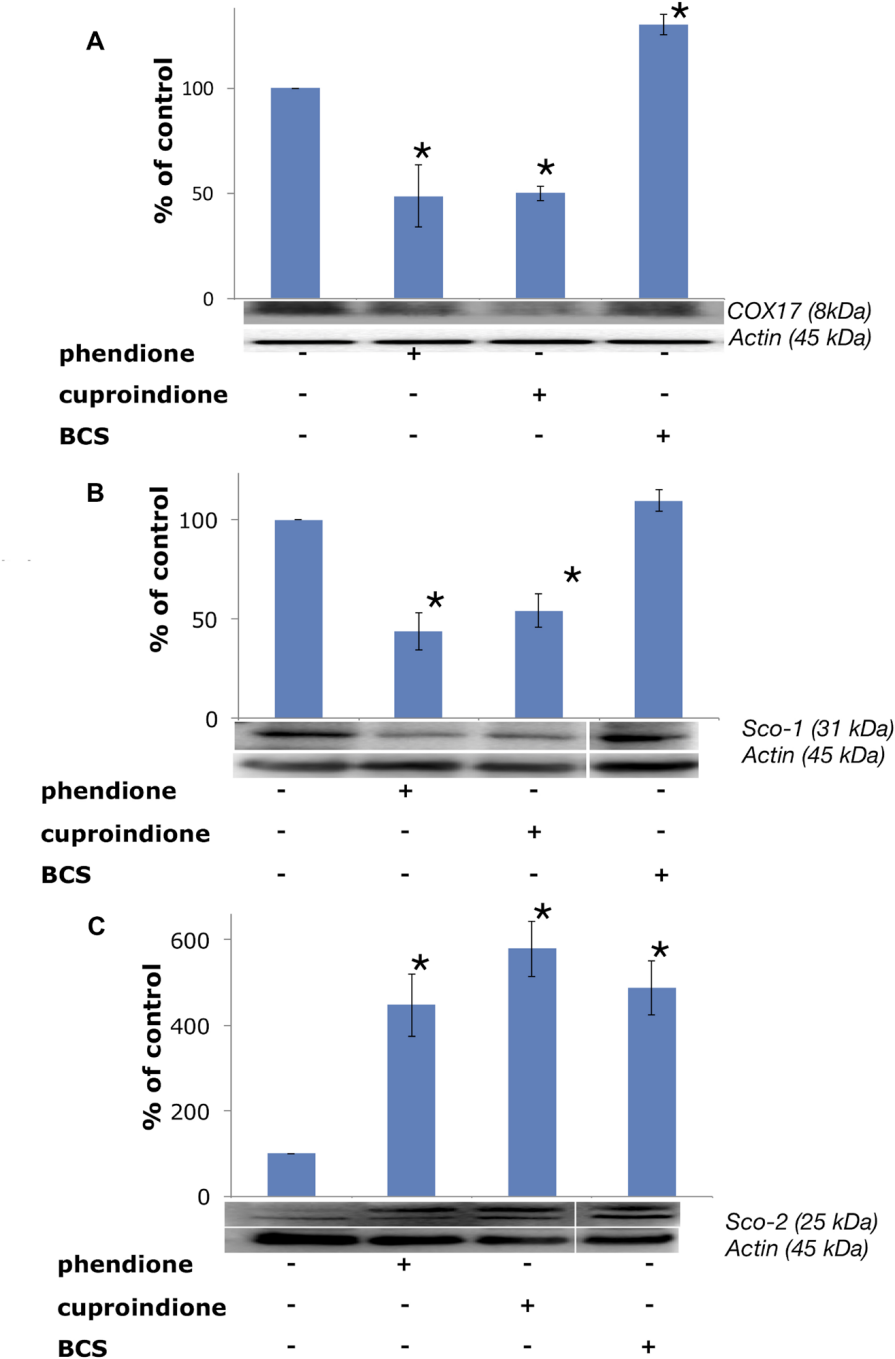
Expression of mitochondrial metallochaperones (**A**) Sco-1, (**B**) COX17 and (**C**) Sco-2 in SH-SY5Y cells after treatment with a phendione, cuproindione IC_50_ concentration and 50 µM BCS for 48 hrs. Results are expressed as mean ± SEM over at least three independent experiments. (^*^p ≤ 0.05 level vs control, One-way Anova).

Following treatment with BCS, COX17 is markedly up-regulated (Figure 10), as expected due to the decreased copper availability to the chaperone resulting from copper complexation to BCS; by contrast, COX17 expression decreases in cells treated with phendione and cuproindione (Figure 10A). To the best of our knowledge, this is the first time that a cytotoxic copper complex has been shown to affect the expression of this CCO chaperone, which raises some questions as to the causes that determine such an effect. The considerations that follow may help answering these questions.

In the first place, the over-expression of COX17, found in some cancer cells, seems to favour proliferation and to maintain CCO efficiency, thus suggesting the use of the chaperone for tumour treatment [179]. Secondly, COX17 is also a newly identified auxiliary factor involved in the control of the architecture of the MICOS complex, a protein complex that is in contact with the outer membrane of mitochondria and is critical to establish and maintain the inner membrane structure. In any case, the COX17-MICOS interaction is promoted by copper ions [180]. Moreover, COX17 forms two independent assemblies, one with SCO1 for the biogenesis of cytochrome *c* oxidase [181], and one with the MICOS complex for mitochondrial membrane organization [182]. Copper regulation is common to both assemblies. The oxidative stress induced by the two ligands investigated in the present study suggests a link between the decreased expression of the copper chaperone and the Bax translocation to the mitochondrial membrane, whose architecture is altered by pore formation. This process is accompanied by the contemporary release of cytochrome C that results in the altered assembly and Sco1 biogenesis assisted by CCO.

The analysis of different colon carcinoma cell lines (Caco-2, HT116, HT29) and cancer cell lines of different tissue origin (MCF7, PC3) showed a transcript-level upregulation of Sco1 [183, 184], suggesting that also this metallochaperone can be a drug target for cancer. The results for Sco1 expression (Figure 10B) are similar to those obtained for COX17, indicating that the down-regulation of this chaperone could contribute to the inhibition of cell proliferation. A link between the oxidative stress induced by our ligands and Sco1 is supported by the reported finding that production of ROS partly induces Fas-mediated cell death of glioma cells by Sco1 down-regulation [185].

Unlike Sco1, Sco2 expression is up-regulated by the cellular treatments with phendione and cuproindione (Figure 10C). This is consistent with the indication that one of the factors for a poor prognosis of patients with breast cancer results also from a low SCO2 expression [186]; noteworthy, exogenous addition of the SCO2 gene to hypoxic cancer cells induces apoptosis and causes a significant regression of tumour xenografts [187]. The complex role played by the two ligands is proved by the involvement of p53 (activated by the oxidative stress) in Sco2 transcription, while the reduced oxidative phosphorylation in p53-null cells is rescued by bringing Sco2 expression back to physiological levels [188]. Our results further stress the dual role of p53 that may serve as a tumour suppressor (activation of Bax) as well as a regulator of mitochondrial aerobic respiration via the modulation of Sco2 synthesis.

## MATERIALS AND METHODS

### Compounds synthesis and characterization

1,10-phenanthroline-5,6-dione and 2,9-dimethyl-1,10-phenanthroline-5,6-dione (Scheme 1) were prepared according to procedures previously reported [189, 190].

Synthesis of [CuCl(phendione)_2_] *ClO*_*4*_*× 3/2H*_*2*_*O*. This complex was prepared as described in the literature [191] with some modifications. A solution of CuCl2 (0.016 gr, 0.118 mmol) dissolved in 5 mL of ethanol was added to a solution containing phendione (0.050 g, 0.237 mmol) in 20 mL of ethanol at 60° C. The mixture was stirred at 60° C for 5 h. After removing any undissolved materials by filtration, LiClO4 (0.253 gr, 2.36 mmol) was added to the filtrate. The obtained green fine precipitated was filtered off, washed with ethanol and then air-dried. Yield: 40%. Anal. Calc. for [CuCl(phendione)_2_]ClO_4_·3/2H_2_O (C_24_H_12_Cl_2_CuN_4_O_8_·3/2H_2_O): C, 44.63; H, 2.34; N, 8.67%. Anal. Found: C, 44.76; H, 2.34; N, 8.58%. ESI-MS: calculated for [CuCl(phendione)_2_]^+^([C_24_H_12_ClCuN_4_ O_4_]^+^): 517.9. Found: m/z (% relative to the base peak) [M^+^]: 517.9 (100). UV-Vis in NaNO_3_ 100 mM: λmax, nm (A): 253 (1.003), 301 (0.370). IR (KBr): 3419.2; 1702.2; 1577.0; 1430.3; 1302.3; 1090.3; 730.3; 936.3; 624.6 cm^-1^.

Synthesis of [Cu(phendione)_3_]*(ClO*_*4*_*)*_*2*_*× 4H*_*2*_*O.* This complex was prepared as described in the literature [192] with some modifications. Phendione (0.050 gr, 0.237 mmol) suspended in 5 mL of ethanol was added to 1.5 mL of pale blue solution containing Cu(ClO_4_)_2_× 6H_2_O (0.029 gr, 0.079 mmol) in ethanol and the resulting green suspension was stirred at r.t. for 30 minutes. The precipitated green solid was filtered off, washed with ethanol and ether, and then air-dried. Yield: 74%. Anal. Calc. for [Cu(phendione)_3_](ClO_4_)_2_·4H_2_O (C_36_H_26_Cl_2_CuN_6_O_18_): C, 44.80; H, 2.72; N, 8.71%. Anal. Found: C, 44.34; H, 2.32; N, 8.44%. ESI-MS: calculated for [Cu(phendione)_3_ + (ClO_4_)_3_ + 2H_2_O]^-^ ([C_36_H_22_Cl_3_CuN_6_O_20_]^-^): 1027.9. Found: m/z (% relative to the base peak): 1027.6 (20). Calculated for [Cu(phendione)_2_ + (ClO_4_)_3_]^-^ ([C_24_H_12_Cl_3_CuN_4_O_16_]^-^): 781.9. Found: m/z (% relative to the base peak): 781.8 (85). Calculated for [Cu(Phendione)_2_(ClO_4_)]^+^([C_24_H_12_ClCuN_4_O_8_]^+^): 581.0. Found: m/z (% relative to the base peak) 581.9 (24). UV-VIS in NaNO_3_ 100 mM: λ_max_, nm (A): 253 (1.003), 301 (0.346). IR (KBr): 3418.5; 1699.4; 1576.3; 1429.1; 1302.1; 1088.9; 730.3; 625.7 cm^-1^.

Synthesis of [Cu(cuproindione)_2_]*(ClO*_*4*_*)*_*2*_·*2H*_*2*_*O.* Cuproindione (0.050 gr, 0.209 mmol) dissolved in 4.5 mL of ethanol was added to 1 mL of a pale blue solution of Cu(ClO_4_)_2_·6H_2_O (0.029 gr, 0.079 mmol) in ethanol; the resulting yellow suspension was stirred at r.t. for 30 minutes. The yellow precipitate was filtered off, washed with ethanol and ether and then air-dried. Yield: 70%. Anal. Calc. for [Cu(cuproindione)_2_](ClO_4_)_2_·2H_2_O (C_28_H_24_Cl_2_CuN_4_O_14_): C, 43.40; H, 3.12; N, 7.23%. Anal. Found: C, 43.14; H, 3.35; N, 6.90%. ESI-MS: calculated for [Cu(cuproindione)_2_-H]^+^ ([C_28_H_20_CuN_4_O_4_]^+^): 539.1. Found: m/z (% relative to the base peak) [M-H] ^+^: 539.3 (100). UV-VIS in NaNO_3_ 100 mM: λ_max_, nm (A): 243 (0.778), 262 (0.998), 303 (0.248), 315 (0.178). IR (KBr): 3436.2; 1698.6; 1590.9; 1315.4; 1090.0; 625.4; 222.8 cm^-1^. The powder samples were stored at −20° C in the darkness. Stock solutions of the compounds were prepared in DMSO (Sigma-Aldrich, St. Louis, MO) at the concentration of 10^–1^ M. Ultrapure milliQ water (18.2 mΩ·cm at 25° C, Millipore) was used throughout. All other chemicals and reagents were of high purity grade and obtained from standard commercial sources. Electrospray ionization mass spectrometry (ESI-MS) was performed with a dual electrospray interface and a quadrupole time-of-flight mass spectrometer (Agilent 6530 Series Accurate-Mass Quadrupole Time-of-Flight (Q-TOF) LC-MS). Elemental analyses were carried out with a Eurovector EA 3000 CHN instrument. IR Spectra were recorded by a Perkin–Elmer Spectrum One and Perkin–Elmer 1600 Series FT-IR spectrophotometers using KBr as solid support for pellets.

### Copper-complexes characterization

#### UV-visible spectroscopy (UV-vis)

UV-vis spectra were recorded with a Varian-Cary 100 UV-Vis Spectrophotometer (Agilent). UV-vis titration experiments were carried out by addition of 1.82 mM aqueous solutions of copper chloride and copper perchlorate (Sigma Aldrich, St. Louis, MO) (from 0.1 equiv. to 1.2 equiv.) to the water solution of phendione or cuproindione (0.065 mM) into the measuring cell containing a known volume (2.8 mL) of the ligand solution.

#### Electron paramagnetic resonance (EPR)

Isotopically pure aqueous solution of ^63^Cu, in the (8·10^–4^ –1·10^–3^) M range of concentration, were obtained from a 5 × 10^–2^ M stock solution of ^63^Cu(NO_3_)_2_ (Sigma Aldrich, St. Louis, MO). EPR spectra were carried out by using a Bruker Elexsys E500 CW-EPR spectrometer driven by a PC running the XEpr program on Linux and equipped with a Super-X microwave bridge, operating at 9.3–9.5 GHz, and a SHQE cavity. The spectra were recorded at 150 K by means of a variable temperature apparatus (ER4131VT). EPR magnetic parameters were obtained directly from the experimental EPR spectra and were calculated from the 2nd and 3rd line to avoid second order effects. The spectra were recorded as an average of 5 scans, microwave frequency 9.344–9.378 GHz; modulation frequency 100 kHz; modulation amplitude 0.2–0.6 mT; time constant 164–327 ms; sweep time 2.8 min; microwave power 20–40 mW; receiver gain 50–60 dB. The solutions were prepared at 1:1 and 1:2 metal-to-ligand ratios in the concentration range 1–1.5 mM. The pH of aqueous solution was adjusted by adding NaOH. 10% of methanol was added to aqueous solutions to enhance spectral resolution at low temperatures.

Distribution diagrams were obtained by using the 2009 version of the Hyperquad Simulation and Speciation program (HySS) [193]. titrations are simulated by specifying a set of titration conditions and calculating the concentrations of each complex species as the titration proceeds. The results are summarized in tabular form and shown graphically.

### Cellular experiments

#### Cell culture maintenance and treatments

Dulbecco’s modified eagle medium (DMEM), Ham’s F-12 medium (F12), streptomycin, L-glutamine, fetal bovine serum (FBS) were provided by Lonza (Verviers, Belgium). Human neuroblastoma (SH-SY5Y line) cells were cultivated not longer than 20 passages in full medium, i.e., DMEM/F12 supplemented with 10% FBS, 2 mM L-glutamine and 100 μg ml^−1^ streptomycin. The cell culture was grown in tissue-culture treated Corning^®^ flasks (Sigma-Aldrich, St. Louis, MO) in humidified atmosphere (5% CO_2_) at 37° C (Heraeus Hera Cell 150C incubator). For the cellular treatments, the day before the experiment cells were seeded at a density of 2·10^5^ cells/mL in full medium on Corning^®^ tissue-culture treated culture dishes (Sigma-Aldrich, St. Louis, MO). Immediately before use, stocks of phendione and cuproindione compounds were diluted (100x) in ultrapure H_2_O and then added to the cells in the culture medium at the desired final concentration (always less than 0.01% v/v of DMSO). For the cell experiments in copper-deprived medium, cells were pre-incubated with 50 μM of extracellular copper chelator BCS for three hrs and, maintaining the same medium, further incubated with the compounds.

#### Cytotoxicity assays

3-(4,5-dimethylthiazol-2-yl)-2,5-diphenyltetrazolium bromide and the hydrated disodium salt of bathocuproinedisulfonic acid (BCS) were purchased from Sigma-Aldrich (St. Louis, MO). The effect of phendione and cuproindione on cell viability was tested at 60–70% of cell confluence by incubation with the compounds with concentrations ranging from 0.01 to 10 × 10^–6^ M for 48 hrs.

In order to calculate the concentration of each compound that produces a 50% cell mortality (IC_50_), the viable cells were quantified by the reaction with 3-(4,5-dimethylthiazol-2-yl)-2,5-diphenyltetrazolium bromide (MTT method, as previously described [194]). After 90 min, the reaction was stopped by adding DMSO, and absorbance was measured at 569 nm (Multiskan Ascent 96/384 Plate Reader). Results were expressed as% of viable cells over the concentration of each compound. The experiments were repeated at least 5 times in triplicate and results expressed as mean ± SEM. The statistical analysis was performed with a one-way Analysis of Variance (ANOVA test, by using the Microcal Origin software, version 8.6).

#### Western blot (WB) analysis

Tris-HCl buffer, ethylenediaminetetraacetic acid (EDTA), Triton X-100, EGTA, nonyl phenoxy polyethoxylethanol (NP40), phenylmethylsulfonylfluoride (PMSF) and bovine serum albumin (BSA) were purchased from Sigma-Aldrich (St. Louis, MO). For the determination of protein amount by WB, cells were incubated at 37° C (in 5% CO_2_ atmosphere) with the compounds. Treatments with IC_50_ concentrations (48 hrs) or 3 × IC_50_ concentrations (90 min) were used for full protein extract or sub-cellular fractions, respectively.

Cells lysates were prepared by cells treatment with RIPA buffer (50 mM Tris-HCl, pH 8.0, 150 mM NaCl, 0.5 mM EDTA, 1% Triton X-100, 0.5 mM EGTA, 1% NP40) containing 2 mM PMSF, an inhibitor of the protease cocktail. Immediately after the addition of the buffer, cells were collected by the scratch method and transferred to Eppendorf tubes (1.5 mL of size, purchased from Sigma-Aldrich, St. Louis, MO) for an incubation on ice for 30 min. After a centrifugation step (10 min at 14,000 r.p.m.) the supernatants were collected and the protein concentration was measured by Bradford’s method using BSA as the standard curve [195]. To prepare sub-cellular fractions (mitochondria and cytoplasm) cell were homogenated in sucrose buffer (0.32 M sucrose, 1 mM EDTA, 10 mM Tris, 1 mM PMSF, pH 7.4) instead of RIPA buffer, and separated by centrifugation according to standard protocols (Abcam).

SDS-PAGE with precast gel (4–20%, BioRad mini-PROTEAN) or with 15% Tricine gel [196] was used to separate proteins lysates or human Atox1 protein oligomers, respectively. Nitrocellulose membranes (Sigma-Aldrich, St. Louis, MO) were used to transfer proteins from the gel. Membranes were incubated with blocking buffer (0.1% Tween20 in tris-buffered saline added with either 5% BSA or 5% non-fat milk, depending on the primary antibody) at room temperature for 1 h, and then incubated with primary antibodies overnight at 4° C. After that, 1 h treatment with horseradish peroxidase-conjugated secondary antibodies was performed.

Primary antibodies used were as follows. From Santa Cruz Biotechnology (Santa Cruz, CA): against PARP-1 (F-2) (code: sc-8007, 1:500 dilution), PARP-1 (B-10) (code: sc-74470, 1:500 dilution), CCS (code: sc-20141, 1:500 dilution), Bcl-2 (code: sc-7382, 1:500 dilution), p53 (code: sc-6243, 1:500 dilution), Bax (code: sc-526, 1:1000 dilution). From Abcam (MA, USA): against GAPDH (code: ab8245, 1:2000 dilution) and CTR1 (code: ab129067, 1:3000 dilution). From Aviva Systems Biology (San Diego, USA): against ATP7A (code: ARP33797, 1:2000 dilution). From Abnova Corporation (Taiwan): polyclonal antibody against Atox1 (code: 15530, 1:2000 dilution) and monoclonal antibody against Atox1 (code: H00000475M01, 1:2000 dilution). From Cell Signaling Technologies Inc. (MA, USA): against Cytochrom C (code: 11940, 1:1000 dilution) and β-Actin (code: 4970, 1:2000 dilution). The secondary antibodies used were from EMD Millipore Bioscience (MA, USA): goat anti-mouse and anti-rabbit IgG horseradish peroxidase-conjugated (AP181P and AP307P, respectively, 1:3000 dilution). Measurements were performed by a ChemiDoc MP Imaging System (BioRad) using enhanced Western Lighting Chemiluminescence Reagent Plus (PerkinElmer (MA, USA). Experiments were repeated independently at least three times and results analysed by one-way ANOVA. The data reported throughout the paper are the mean values ± SEM and to the most representative membrane images.

### Mitochondrial membrane potential measurements

5′,6,6′-tetrachloro-1,1′,3,3′-tetraethylbenzimidazolylcarbocyanine iodide (JC-1) was purchased from Molecular Probes (ThermoFisher Scientific, MA, USA) [197]. In order to determine the mitochondrial membrane potential, cells were treated with 3 × IC_50_ concentration of the compounds for 90 min, further incubated with 2 μg/mL JC-1 (in the dark at 37° C) for 30 min, and washed twice with PBS to remove the unbound dye. A fluorescence plate reader (Varioskan^®^ Flash Spectral Scanning Multimode Readers, Thermo Scientific (excitation wavelength = 488 nm) was used to monitor the fluorescence intensities for JC-1 molecules, either in monomer (emission wavelength = 530 nm) and in aggregated (emission wavelength = 595 nm) forms, respectively. Experiments were carried out simultaneously in by using eight wells for each compound; at least three independent experiments were performed. Results were analysed by one-way ANOVA expressed as fluorescence ratio (I = 595/530 nm) mean values ± SEM. For the detection of mitochondrial O^2•−,^ after the treatment with phendione or cuproindione (at 3 × IC_50_ concentrations) for 30 minutes, cells were stained with superoxide probe MitoSOX (ThermoFisher Scientific, MA, USA). Hoechst 33342 solution was used for normalizing data to the actual cell number for each well. The fluorescence intensities were recorded at excitation/emission wavelengths of 510/580 nm for MitoSOX and 361/497 for Hoechst 33342 solution, respectively. In addition, in this case, experiments were carried out simultaneously in by using eight wells for each compound; at least three independent experiments were performed. Results were analysed by one-way ANOVA and expressed as mean values ± SEM.

### Glutathione (GSH) levels determination

The changes of total intracellular GSH in treated with respect to untreated cells was assessed by using Glutathione Detection Assay Kit (Fluorometric, ab65322, Abcam, MA, USA). Cells were incubated with phendione or cuproindione at IC_50_ concentration for 24 hrs, and lysates obtained by addition of the lysis buffer provided in the kit. After an incubation on ice for 30 min, the lysates were centrifuged (10 min at 14,000 r.p.m.), the supernatants collected and processed according to the kit protocol, to eventually measure fluorescence (excitation/emission wavelengths = 380/461 nm).

### Glutathione redox state determination

Cells were incubated with phendione or cuproindione at 3 × IC_50_ concentration for 90 min, then trypsinized and counted to obtain a cell suspension of 8·10^5^ cells/mL. In order to evaluate the ratio of GSH/GSSG we used the detection assay kit from Abcam (ab138881, Abcam, MA, USA). Lysates were obtained by adding PBS with 0.5% of NP-40. Cells were homogenized by pipetting and centrifuged (10 min at 14,000 r.p.m.). Supernatants were treated by deproteinizing Sample Preparation Kit–TCA (ab204708, Abcam, MA, USA) and were then collected and processed according to the kit protocol (excitation/emission wavelengths = 490/520 nm).

### Scanning confocal microscopy (LSM) analyses

For confocal microscope imaging, cells were seeded on glass bottom dishes (WillCo-dish^®^, Willco Wells, B.V.) with 12 mm of glass diameter at a density of 1.5 × 10^4^ cells per dish, with complete medium for 24 hrs until cellular adhesion was attained. Cells were incubated with phendione and cuproindione (at 3 × IC_50_ concentrations) for 90 min in complete medium without FBS. Fifteen minutes before stopping incubation, Hoechst 33342, MitoTracker™ Deep Red FM (Thermo Fisher) and Coppersensor-1 (CS1) (1 μM, Cu(I) selective probe were added, for the staining of nuclei, mitochondria, and intracellular copper, respectively [161, 162, 198]. Cells were then fixed with high purity 2% paraformaldehyde in PBS (pH = 7.3). Images were acquired with an Olympus FV1000 confocal microscope equipped with the following UV/visible lasers: diode laser (50 mW, λex = 405 nm), Argon laser (20 mW, jλex=488 nm) and HeNe(R) laser (1 mW, λex = 633 nm) for the imaging of nuclei (blue channel), monovalent copper by CS1 (green channel) and mitochondria (red channel), respectively. An oil immersion objective (60xO PLAPO) was used; the detector gain was fixed at a constant value, and the emitted light was detected in sequential mode. All images were taken randomly throughout the area of the cell culture wells. The image analysis was carried out using Huygens Essential software (by Scientific Volume Imaging B.V., The Netherlands). The statistical analysis was performed with a one-way ANOVA.

### *Atox1* oligomerisation

Atox 1 protein was expressed and purified according to previously reported procedures [199]. For oligomerisation, Atox1 samples (2.2·10^–5^ M) were incubated in 0.01 PBS with phendione or cuproindione (1:1 mole ratio) both with and without CuSO_4_ (1:1:1 mole ratio) for 20 hrs. Parallel experiments were run in the presence of H_2_O_2_ (2.5-fold excess with respect to the cystein residues of the Atox1) [200]. Atox1 oligomeric species were separated on 15% Tricine gel in non-reducing conditions, transferred to nitrocellulose membranes and then incubated with primary antibodies (polyclonal anti-Atox1 antibody (code: 15530, 1:2000 dilution) overnight at 4° C (For a more detailed description see the WB analysis section reported above).

## CONCLUSIONS

Phendione and cuproindione bind and transport inside the cell the copper ion present in the culture medium, giving rise to oxidative stress. Assuming that the two carbonyl groups present in phendione and cuproindione do not alter the copper affinity constants of the parent ligands (2,9-dimethyl-1,10-phenanthroline-5,6-dione and 2,9-dimethyl-1,10-phenanthroline), speciation results indicate that the bis-chelate complex species are mainly responsible for the cellular response. In three cases (increase of IC_50_ in SH-SY5Y cells (Figure 3), increase of cleaved/full PARP-1 ratio (Supplementary Figure 2), and downregulation of anti-apoptotic Bcl-2 (Supplementary Figure 3), the addition of BCS nullifies the cuproindione-induced effect, reflecting the different copper affinity of the two investigated ligands.

The binding of the metal ion to cuproindione and phendione changes copper chaperone speciation and alters copper redox homeostasis, through the oxidation of thiol groups. These copper complex species can also cause DNA-strand break by direct interaction with DNA (non-intercalative binding to the minor groove of DNA[80]). We found several possibly interconnected effects, i.e., the decrease of the GSH/GSSG ratio (Figure 4), the depolarization of mitochondrial membrane and production of ROS (Figure 5), the decrease of monomeric Atox1 (Figure 8) and the decrease of GSH levels (Supplementary Figure 4). Moreover, phendione and cuproindione up regulate differently p53 expression, depending on their different oxidative nuclease ability, (Figure 6). In addition, the p53 transcription factor is not only the key driver of mitochondria apoptosis but also participates in the mitochondria metallostasis both by altering the intracellular location of metal ion and by controlling the synthesis of Sco-2 that, in turn, increases ROS levels.

Usually, copper homeostasis has been investigated focusing on a single component of the metallostasis network; instead, our investigation involves several of the factors controlling the metallostasis. This is the only approach that allows for an exhaustive understanding of how the communication within the dynamic metallome drives the final decisions made by the cells.

## SUPPLEMENTARY MATERIALS FIGURES


