# Supplementary material for: Cytotoxic phenanthroline derivatives alter metallostasis and redox homeostasis in neuroblastoma cells
Source: Oncotarget. 2018 Nov 20;9(91):36289–316. doi: 10.18632/oncotarget.26346 (PMC6284747; doi:10.18632/oncotarget.26346)
Supplement: Supplementary file 1 [file oncotarget-09-36289-s001.pdf]

Cytotoxic phenanthroline derivatives alter metallostasis and redox homeostasis in neuroblastoma cells

SUPPLEMENTARY MATERIALS

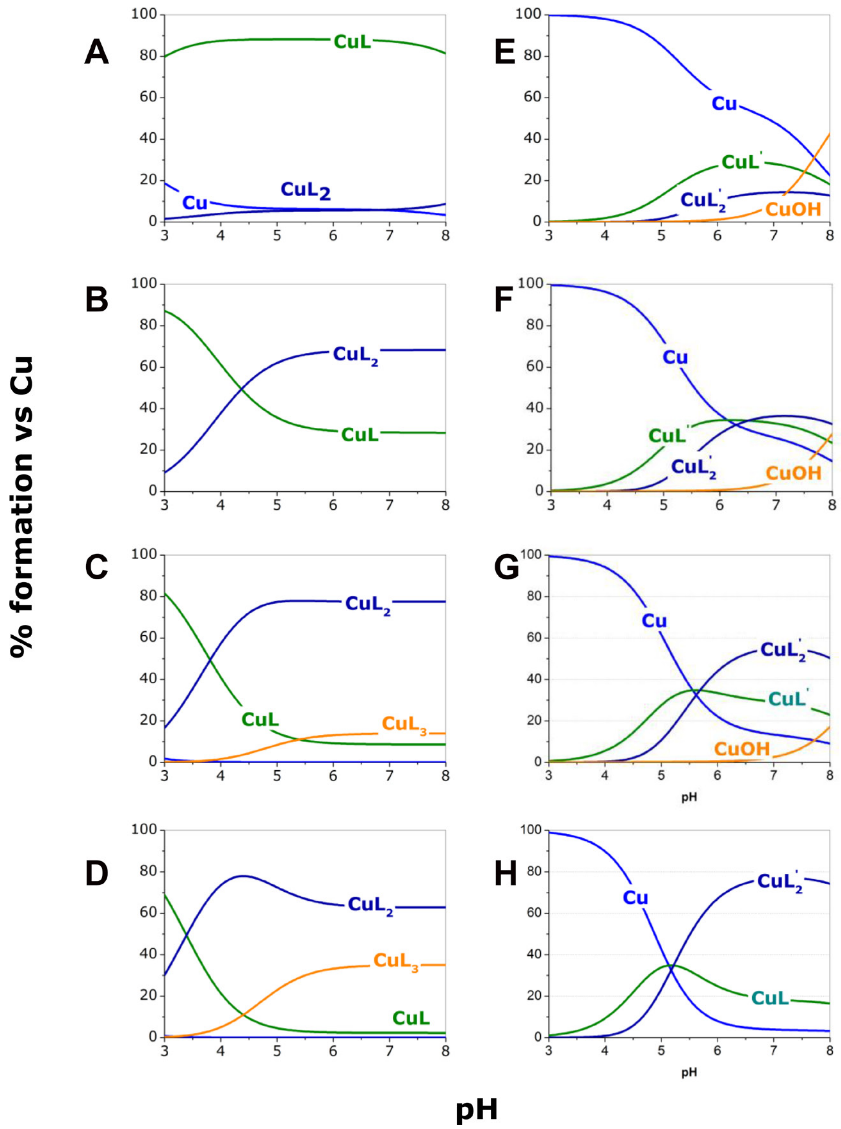

Supplementary Figure 1: Species distribution computed for phendione (L) and cuproindione (L'). In all species distribution diagrams Cu2+ is 1.9 µM; in (**A–D**) as well as in (**E–H**) the ligand is 1,2,3 and 5 times the copper concentration, respectively. Species forming in concentrations<5% are omitted for clarity.

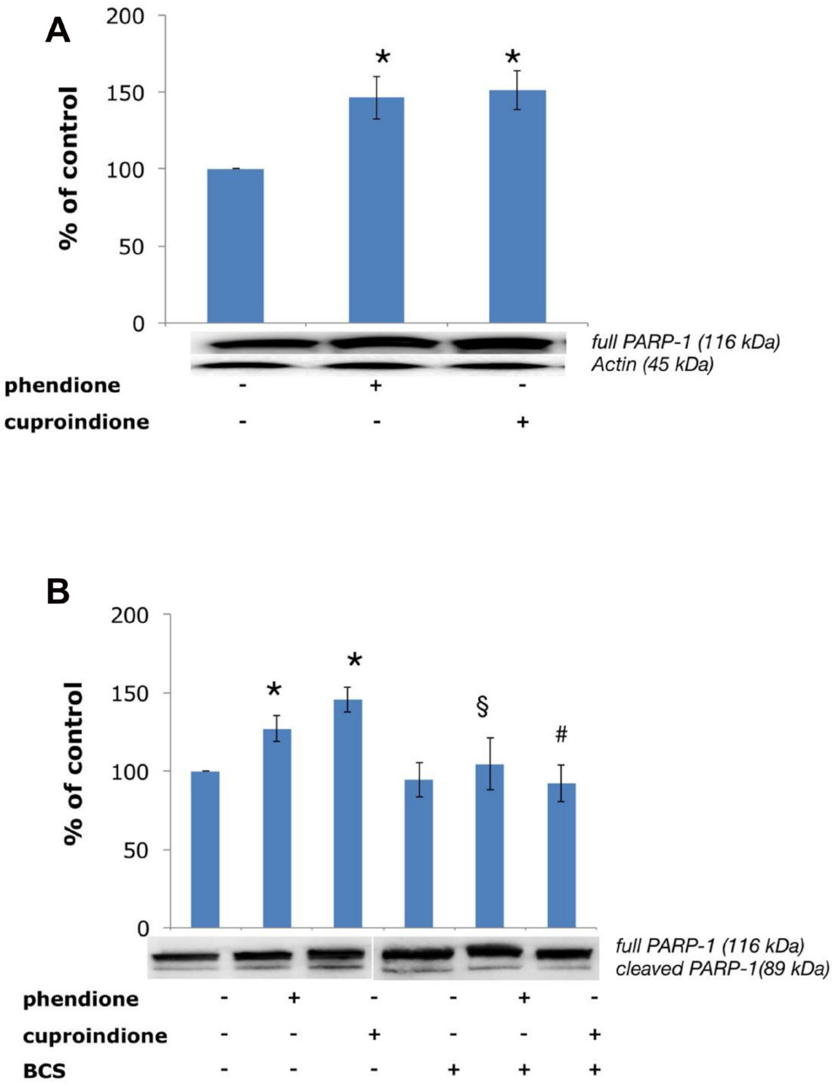

Supplementary Figure 2: Expression and cleavage of PARP-1 in SH-SY5Y cells after treatment with phendione or cuproindione or pre-incubation with BCS. Expression and cleavage of PARP-1 in SH-SY5Y cells after treatment with 0.1 µM (**A**) or IC50 concentration (**B**) of phendione or cuproindione for 48 hrs either in the basal culture medium or after pre-incubation (3 hrs) with 50 µM BCS. The data in (B) represent the percent of cleaved PARP-1 (89 kDa)/full PARP-1 (116 kDa) ratios. Results are expressed as mean ± SEM over at least three independent experiments ($^{*}p \leq 0.05$ level vs phendione, $^{\S}p \leq 0.05$ level vs phendione, $^{\#}p \leq 0.05$ level vs cuproindione; One-way Anova)

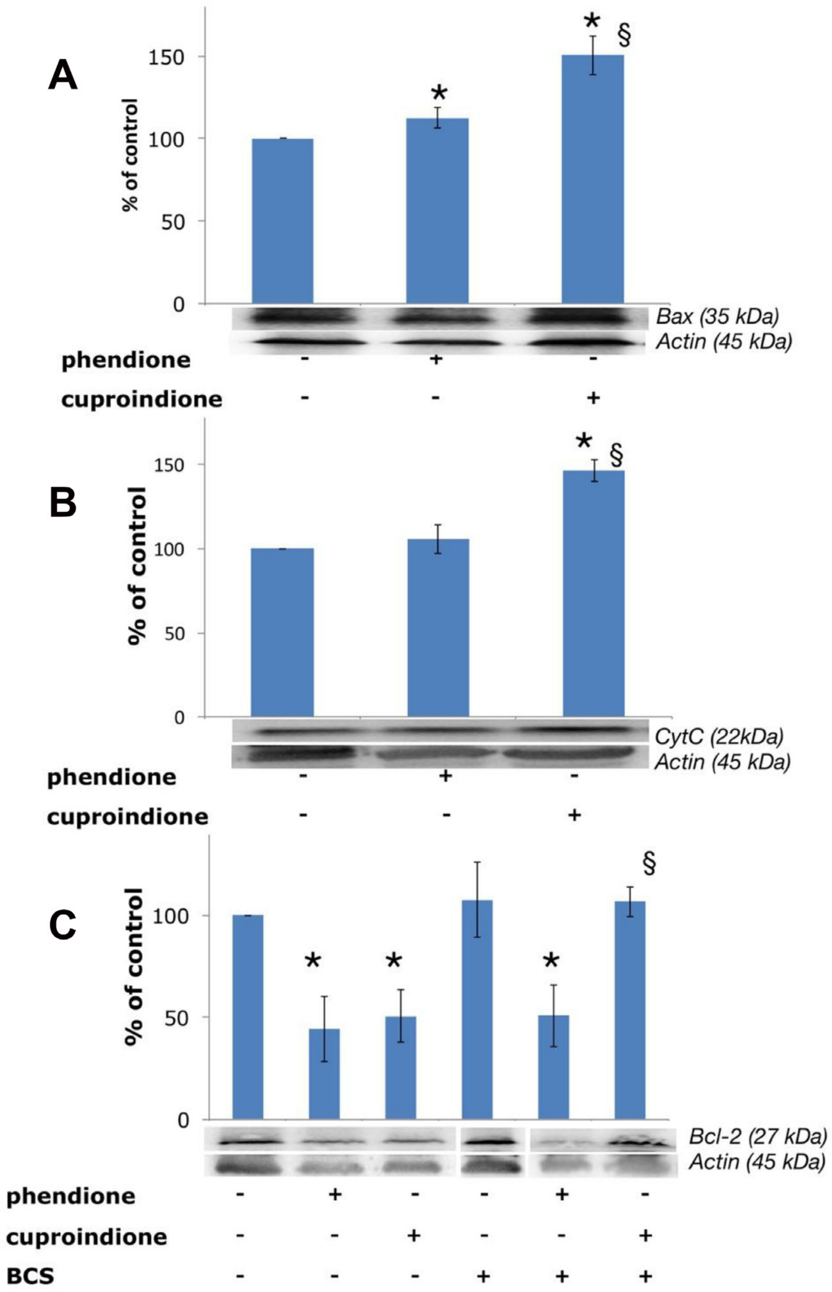

Supplementary Figure 3: Levels of Bax in the mitochondrial fractions and of cytochrome C in the cytoplasm fractions for cells treated with phendione or cuproindione. Levels of Bax in the mitochondrial fractions (**A**) and of cytochrome C in the cytoplasm fractions (**B**) for neuroblastoma cells after a 90-min treatment with a phendione and cuproindione 3 × IC_{50} concentration. Expression of Bcl-2 (**C**) after a 48-hrs treatment with IC50 concentration of phendione and cuproindione in the absence and in the presence of 50 μM BCS. Representative WBs of the extracts are shown. Results are mean ± SEM over at least three independent experiments ($^{*}p \leq 0.05$ level with respect to control, $^{§}p \leq 0.05$ level with respect to phendione; One-way Anova).

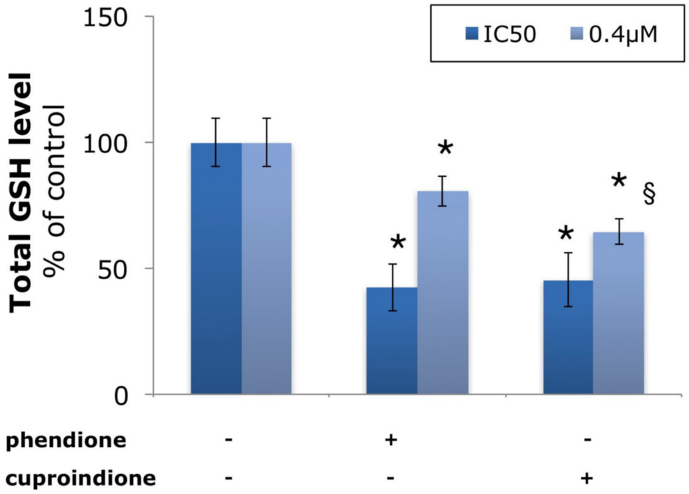

Supplementary Figure 4: Effect of phendione or cuproindione on total GSH level in cells 24-hrs treated with IC50 or 0.4 µM concentrations. Results are expressed as mean ± SEM from triplicate experiments and normalized with respect to the control untreated cells. ($^*p \leq 0.05$ level with respect to control; $^{\S}p \leq 0.05$ level with respect to phendione; One-way Anova).

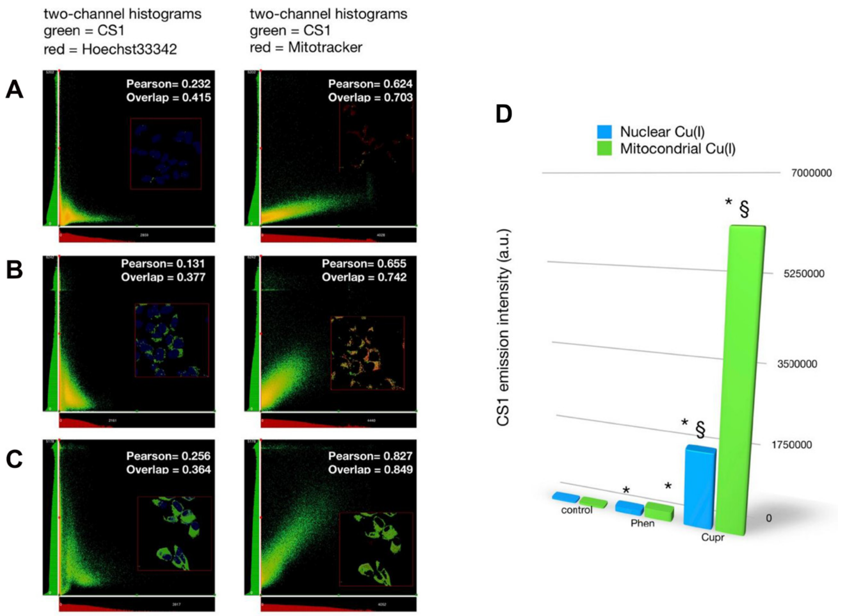

Supplementary Figure 5: Quantitative results of co-localization and copper uptake in the nuclei and mitochondria of cells treated with phendione or cuproindione. Quantitative results of co-localization (**A**–**C**) and copper uptake (**D**) in the nuclei and mitochondria of untreated neuroblastoma cells (A) and in cells treated for 90 min with 4.4 M phendione (B) or 2.2 M cuproindione (C). Results in (D) are expressed as mean ± SEM over at least three independent experiments. ($^*p \leq 0.05$ level with respect to control, $^{\S}p \leq 0.05$ level with respect to phendione; One-way Anova).
